# A new mechanistic model for individual growth applied to insects under ad libitum conditions

**DOI:** 10.1371/journal.pone.0309664

**Published:** 2024-09-04

**Authors:** Karl Mauritsson, Tomas Jonsson

**Affiliations:** 1 Ecological Modelling Group, School of Bioscience, University of Skövde, Skövde, Sweden; 2 Ecological and Environmental Modeling, Department of Physics, Chemistry and Biology, Linköping University, Linköping, Sweden; Universidade Federal de Lavras, BRAZIL

## Abstract

Metabolic theories in ecology interpret ecological patterns at different levels through the lens of metabolism, typically applying allometric power scaling laws to describe rates of energy use. This requires a sound theory for metabolism at the individual level. Commonly used mechanistic growth models lack some potentially important aspects and fail to accurately capture a growth pattern often observed in insects. Recently, a new model (MGM–the Maintenance-Growth Model) was developed for ontogenetic and post-mature growth, based on an energy balance that expresses growth as the net result of assimilation and metabolic costs for maintenance and feeding. The most important contributions of MGM are: 1) the division of maintenance costs into a non-negotiable and a negotiable part, potentially resulting in maintenance costs that increase faster than linearly with mass and are regulated in response to food restriction; 2) differentiated energy allocation strategies between sexes and 3) explicit description of costs for finding and processing food. MGM may also account for effects of body composition and type of growth at the cellular level. The model was here calibrated and evaluated using empirical data from an experiment on house crickets growing under ad libitum conditions. The procedure involved parameter estimations from the literature and collected data, using statistical models to account for individual variation in parameter values. It was found that ingestion rate cannot be generally described by a simple allometry, here requiring a more complex description after maturity. Neither could feeding costs be related to ingestion rate in a simplistic manner. By the unusual feature of maintenance costs increasing faster than linearly with body mass, MGM could well capture the differentiated growth patterns of male and female crickets. Some other mechanistic growth models have been able to provide good predictions of insect growth during early ontogeny, but MGM may accurately describe the trajectory until terminated growth.

## 1. Introduction

### 1.1 Metabolic theories

In many respects, the individual organism is a special level of organization. From an evolutionary perspective, selection occurs at the level of the individual, while from an ecological perspective, patterns and processes in populations, communities and ecosystems all result from activities of and interactions between individuals. Since energy fuels these activities, how individuals acquire and use energy influences many other levels in biology. Accordingly, metabolic theories in ecology interpret processes at different ecological levels through the lens of metabolism. Much work has been published within the framework known as the *metabolic theory of ecology* (MTE) [1], which is based on allometric scaling and temperature dependence of metabolic rate *R* for individual organisms, allowing inference about broad patterns in higher level ecological patterns to be made [2–8]. The master equation in MTE can be written as:

$$R=\alpha W^{\beta }\cdot e^{\varepsilon (T-T_{0})/(\kappa TT_{0})}.$$ (1)

Here *W* is body mass, *T* is absolute temperature, *α* is a normalization constant, *β* is an allometric exponent, *ε* is the activation energy, *κ* is Boltzmann’s constant and *T*_0_ is a reference temperature that sets the temperature-dependent factor ($e^{\varepsilon (T-T_{0})/(\kappa TT_{0})}$) to 1 at *T* = *T*_0_. Metabolic rate here usually means *resting metabolic rate*, the metabolic rate of a non-feeding inactive organism. The *total* (or *active*) *metabolic rate* of a free-living organism, spending energy on foraging and other activities, is usually significantly higher [1].

Although extensively used, from predicting population growth rates [9, 10] and carrying capacities [11] via parameterization of food web models [12–15], to explanation of food chain length and trophic position of consumers [16], MTE has been widely criticized for its weak mechanistic basis [17–19] and debatable support for its alleged generality. Building on a ¾ interspecific allometric scaling of metabolic rate across species, originally claimed to originate from limitations of a resource distribution network [20], the support for this explanation is weak and the generality of the ¾ exponent contested [17, 21–24]. Contrary to the assumption of MTE, many studies have shown that the effects of body mass and temperature on resting metabolic rate are not independent [21, 25, 26] and MTE has been criticized for ignoring that metabolic rates often respond to biological processes under the influence of information processing, that manages energy allocation as adaptive responses to environmental factors [27, 28]. As a result, MTE now can be said to be more of a phenomenologically based theoretical framework where metabolic rates seem to follow a power relationship with body mass (but where there may be systematic taxonomic variation in the value of *β*). Thus, to make this framework more mechanistically grounded and better understand the deviations of individual species from a general interspecific relationship, an empirically validated mechanistically based metabolic model of the growth of individuals is needed.

Many suggestions have been put forward to explain observed apparent allometric scaling of metabolic rate [28–30], including views where metabolic rates are limited by resource distribution networks [20, 31], fluxes across exchange surfaces [32, 33], composition of body components with different metabolic activity [34, 35], resource demand of different whole-body processes [36, 37], and effects of somatic growth by cell enlargement vs. cell division [31]. *Growth models* try to capture processes of this kind that are relevant for one or several life stages at the individual level. The underlying theory for such a model is thus a foundation for metabolic theory. Phenomenological growth models propose mathematical functions that can provide a good fit to empirical curves, like the famous growth model by Gompertz [38] or more recent innovations [39, 40]. However, they provide little mechanistic insight into affecting factors during growth. Mechanistic growth models instead are based on underlying mechanistic assumptions combined with an energy or mass balance that describes growth as the net result of contribution and consumption processes. Here, a recently proposed such model [41] is analysed and validated, resulting in a description that better replicates observed growth trajectories in an insect.

### 1.2 Growth models

Growth models are often expressed as a *growth equation*, a differential equation that relates the instantaneous growth rate *dW*/*dt* (increase of body mass *W* per unit of time *t*), to the current body mass *W*(*t*). Under ad libitum conditions many mechanistic growth models so far have the same general mathematical structure, in which the growth rate can be described as the difference between two allometric terms:

$$\frac{dW}{dt}=aW^{b}-cW^{d}.$$ (2)

We call this the Generalized Standard Growth Model (GSGM). The first term (*aW*^*b*^) represents a contribution process in some form, while the second (*cW*^*d*^) summarizes various types of consumption processes or costs. Depending on the assumptions made and mechanisms thought to be important and included, the detailed formulation of contribution and consumption processes differ among growth models. However, three major groups of mechanistic growth models (see Note SI1 in S1 File) can be distinguished based on how contribution and consumption processes are interpreted and described (Table 1).

**Table 1:** Major groups of common mechanistic growth models under ad libitum conditions, with specification of contribution and consumption processes (Eq (2)) and applied values of the allometric exponents *b* and *d*.

Model type	Contribution process (*aW*^*b*^)	Consumption process (*cW*^*d *^)	*b*	*d*
AnaCat^1^	Anabolism	Catabolism	2/3 (Pütter) adjustable (vB & Pauly)	1 (Pütter & vB) ≤1 (Pauly)
DEB^2^	Assimilation	Maintenance	2/3	1
OGM^3^	Anabolism	Maintenance	3/4	1

Note

1: Anabolism-Catabolism based growth models [42–44]

2: Dynamic Energy Budget growth models [45–47]

3: Ontogenetic Growth Models [48–50]

Shortcomings with commonly applied mechanistic growth models so far (e.g. von Bertalanffy [43], DEB [45], OGM [49]) are that they often fail to fully capture growth in insects [41, 51] and aquatic invertebrates [52, 53] and are ‘simple’ in the sense that they focus on a single mechanism assumed to be important. However, the allocation of available energy to different components of an animal’s energy budget, including activity, maintenance, growth and reproduction, is known to be strongly dependent on the ecology and life-history of species [54] and metabolism is likely a result of diverse adaptations under different ecological constraints, including several interactive and overlapping processes that affect growth [27, 55–57].

A more detailed and flexible growth model that considers several potentially relevant aspects of regulated metabolism and growth may however be able to address this and better describe growth of insects (and other organisms). Such a model should be able to capture the following aspects: 1) deviations from allometric power scaling of metabolic components; 2) regulated metabolism under food restriction; 3) costs of finding and processing food; 4) effects of body composition on costs for growth and maintenance; 5) differences between somatic and reproductive tissue regarding costs for growth and maintenance; 6) sex differences in allocation strategies; 7) effects of cellular growth patterns on costs for growth and maintenance. The *Maintenance-Growth Model* (MGM, [41] is a framework that includes all of these aspects, but can be simplified for specific situations depending on the biology of the organism. MGM has some similarities with the major growth models summarized above (Table 1) in that it under ad libitum conditions and a number of simplifying assumptions (mainly concerning maintenance costs, see Mauritsson and Jonsson [41]), also can be written in the general form of Eq (2). In less simplified cases, MGM takes account of body composition, cellular growth patterns and a life-history determined trade-off between maintenance costs and growth-related costs in ways that preclude it from being written in the general form of Eq (2).

**Fig 1:**
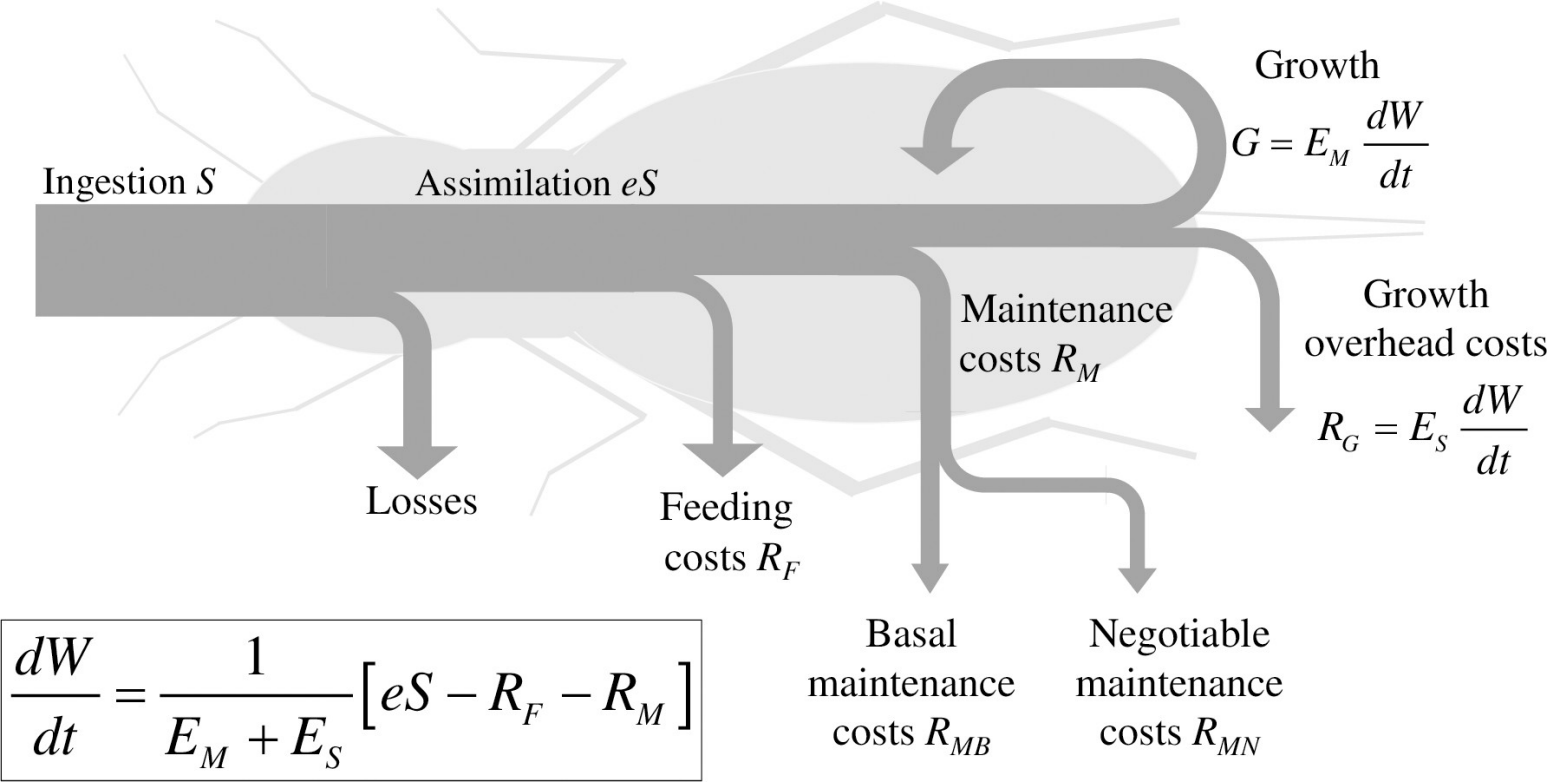
Energy balance and resulting growth equation of the Maintenance-Growth Model (MGM). Arrows represent fluxes of energy through a growing (non-reproducing) animal. Some of the energy ingested as food (*S*) disappears as losses through egestion or excretion. The assimilated energy (*eS*) is distributed between feeding costs (*R*_*F*_), maintenance costs (*R*_*M*_), growth overhead costs (*R*_*G*_) and energy becoming bounded in reproductive and somatic biomass (*G*). Maintenance costs are divided between basal (*R*_*MB*_) and negotiable (*R*_*MN*_) maintenance costs.

A general formulation of MGM is expressed by the following growth equation (see Note SI2 in S1 File for the derivation and Fig 1 for an illustration of the underlying energy balance):

$$\frac{dW}{dt}=\frac{1}{E_{M}(W)+E_{S}(W)}[eS(S_{\lim},W)-R_{F}(S)-R_{M}(\varphi ,W)].$$ (3)

The energy density of newly synthesised body tissue *E*_*M*_ may vary with the body mass *W* if the body composition changes during growth, but it is otherwise constant. The specific growth overhead cost *E*_*S*_ is the energy lost as heat per unit of synthesised body mass (and may depend on *W*). The assimilation efficiency *e* is a constant related to food type and quality. The ingestion rate *S* is a function of food availability *S*_*lim*_ and body mass *W*. Feeding costs *R*_*F*_ are metabolic costs for searching and processing food, assumed to be a function of the ingestion rate *S*. Maintenance costs *R*_*M*_ include various homeostatic processes and is further divided into non-negotiable *basal* maintenance costs *R*_*MB*_ that are necessary to keep the animal alive and *negotiable* maintenance costs *R*_*MN*_ that keep the animal in good shape. Basal maintenance costs constitute a bare-minimum requirement for survival, whereas ‘negotiable’ maintenance costs can be ‘tuned down’ in order to save energy, including defence, repair, immune responses and maintenance of somatic tissues that can be reduced in the short term without serious lethal danger (e.g. fat or muscle tissues). Both basal and negotiable maintenance costs increase with body mass *W*, but *R*_*MN*_ may also be up- or downregulated in response to the relative food acquirement *φ* and is affected by *W* in a way that is dependent on the life history strategy of the animal. The relative food acquirement *φ* measures the level of food availability as the ratio between realised ingestion rate (*S*) and the ingestion rate that the animal would have under ad libitum conditions (*S*_*max*_) at its current body mass; *φ* = *S* /*S*_*max*_. Specifications of all these MGM components under ad libitum conditions (*φ* = 1), leading to the version of MGM analysed here (Eq (4)), are described in Note SI3 in S1 File.

To calibrate and evaluate the model, empirical data was collected from an experiment on house crickets (*Acheta domesticus*), growing under ad libitum conditions. We find that MGM is able to capture a number of aspects that are not covered by common mechanistic growth models and predict the observed growth patterns in an insect, from birth to ultimate size, that GSGM (Eq (2) with *d* ≤ 1) is unable to accurately describe [41, 51].

## 2. Materials and methods

### 2.1 Experimental setup

42 newly hatched nymphs of house crickets (*Acheta domesticus*) were collected from a laboratory culture (initially purchased from a zoological supplier), weighted and reared individually in plastic boxes under ad libitum conditions and an ambient temperature of 28.6 ± 0.9°C for 72 days. A controlled amount of food (a mixture of pellets for guinea pigs and rats) was provided twice a week, when the weight of the cricket, as well as food and faecal material remaining, was measured. Age at imago emergence was recorded for each individual. At some occasions, faeces were separated from remaining food and weighted separately (in order to estimate the relative egestion rate). At the end of the experiment useful data for 35 individuals (14 males, 21 females) had been produced. Moisture uptake in food under current laboratory conditions was estimated (required to accurately calculate food consumption from collected data). More details on the experimental setup are found in Note SI4 in S1 File.

### 2.2 Model simplifications for application of MGM to house crickets

See Note SI2 in S1 File for a general derivation of MGM type growth models, and Note SI3 in S1 File for details and specification of MGM components under ad libitum conditions. A number of simplifying assumptions were made to limit the number of free parameters in the application of the growth model (Eq (3)) to collected data for house crickets. First, constant fractions of lipids, carbohydrates and proteins in somatic and reproductive tissue were assumed, resulting in the same constant biomass energy density *E*_*M*_ for both types of tissue (Note SI3.1 in S1 File). To obtain a continuous growth rate at imago emergence (according to Eq (3)), the same specific overhead cost for somatic and reproductive growth were assumed, resulting in a constant specific growth overhead cost *E*_*S*_ (Note SI3.2 in S1 File). Second, the specific overhead cost for somatic cell division and cell growth were assumed to be the same. Third, basal maintenance was assumed to be independent of cell size, resulting in constant mass-specific basal maintenance costs *γ*_*BS*_ and *γ*_*BR*_ for somatic and reproductive tissue, respectively. See Note SI9.3 in S1 File for the consequences of deviating from these assumptions. Since ad libitum conditions were considered, the relative food acquirement was put to *φ* = 1. The ad libitum ingestion rate *S* = *S*_*max*_ was described by a power allometry until imago emergence (*W* ≤ *W*_*mat*_) and thereafter by a more complicated function of body mass (Note SI3.3 in S1 File). Feeding costs *R*_*F*_ were described by a piece-wise linear function of *S* (Note SI3.4 in S1 File). Maintenance costs *R*_*M*_ were described differently for males and females, accounting for different allocation strategies regarding somatic and reproductive tissue (Note SI3.5 in S1 File). All of this resulted in the following final version of the growth model:

$$\frac{dW}{dt}=\frac{1}{E_{M}+E_{S}}[eS-R_{F}(S)-R_{M}],\{\begin{array}{l} S=\{\begin{array}{lll} \alpha W^{\beta } & , & W\leq W_{mat}\\ \alpha W_{mat}^{\beta }+k[\frac{W^{*}(W^{\beta }-W_{mat}^{\beta })}{\beta }-\frac{W^{1+\beta }-W_{mat}^{1+\beta }}{1+\beta }] & , & W\geq W_{mat} \end{array}\\ R_{F}(S)=\{\begin{array}{lll} k_{F1}\cdot S & , & S\leq S_{1}\\ k_{F1}\cdot S_{1}+k_{F2}\cdot (S-S_{1}) & , & S\geq S_{1} \end{array}\\ R_{M,males}=\frac{\gamma_{B}W}{1-a_{N}W}\\ R_{M,females}=\{\begin{array}{ll} \frac{\gamma_{BS}W}{1-a_{N}W} & ,W\leq W_{mat}\\ \frac{\gamma_{BS}W_{mat}}{1-a_{N}W_{mat}}+\frac{\gamma_{BR}(W-W_{mat})}{1-a_{N}(W-W_{mat})} & ,W\geq W_{mat} \end{array} \end{array}.$$ (4)

**Fig 2:**
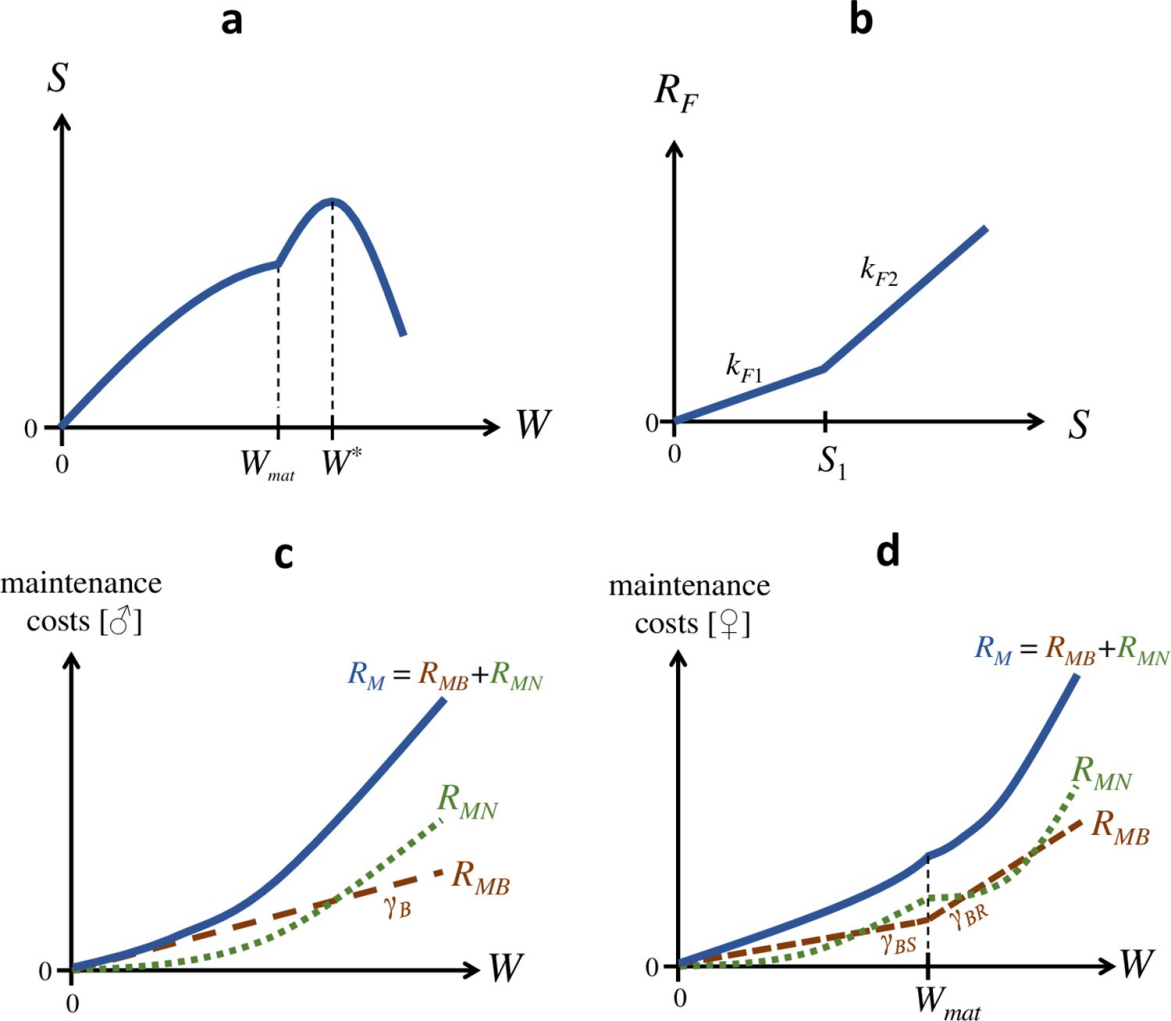
Components of MGM for house crickets under ad libitum growth. **a**) Ingestion rate (*S*) as a function of body mass (*W*), initially a power function that breaks at maturity (*W* = *W*_*mat*_). **b**) Feeding costs (*R*_*F*_) as a function of ingestion rate (*S*), shaped as a ‘hockey-stick’. **c-d**) Maintenance costs (*R*_*M*_) as a function of body mass (*W*) for (**c**) males and (**d**) females, including basal maintenance costs (*R*_*MB*_) and negotiable maintenance costs (*R*_*MN*_). In males, basal maintenance costs increase linearly with total body mass. In females, basal maintenance costs increase linearly with somatic body mass until maturity (*W* = *W*_*mat*_) and then increase linearly with reproductive body mass. Negotiable maintenance costs increase superlinearly with body mass in both sexes.

See Fig 2 for explanatory illustrations of the model components as described by Eq (4). Analysis and validation of this version of MGM first required estimations of model parameters. This was a four-step process where parameters were (*i*) obtained from literature, (*ii*) estimated directly from experimental data, (*iii*) estimated from experimental data with statistical methods applied to MGM descriptions of specific model components, or (*iv*) estimated indirectly using ‘inverse model optimization’. Required model assumptions and parameter estimations for these three steps are described below. Once values for all parameters had been obtained (SI9 Table in S1 File), the performance of the model was evaluated by comparing its predictions to experimental observations.

### 2.3 Pre-experimental parameter estimations from literature

A number of model parameters could be obtained prior to the experiment, estimated from the literature. This included the ‘primary MGM parameters’ biomass energy density *E*_*M*_ and assimilation efficiency *e* (SI9 Table in S1 File), as well as the ‘associated parameters’ resting metabolic rate normalisation constant *a*_*R*_ and allometric exponent *b*_*R*_, which in combination with experimental data were used to estimate other MGM parameters in a later step. To convert ingestion rates from a mass basis (mg/day) to an energy basis (J/day), the food energy density *E*_*f*_ was required. Details on calculations of food energy density, biomass energy density, assimilation efficiency and resting metabolic rate parameters are provided in Note SI5 in S1 File.

### 2.4 Model parameters estimated from experimental data

Prior to fitting the model to the experimental data, a number of model parameters could be estimated without model optimization. That is, life-history parameters (such as birth mass and mass at maturity, SI9 Table in S1 File) were obtained directly from growth and ingestion measurements, ingestion rate parameters (*α*, *β*, *k*_,_ W*, SI9 Table in S1 File) were estimated with mixed effects models directly from data collected in the experiment, while feeding costs parameters (S1, *k*_*F*1,_ *k*_*F*2_, SI9 Table in S1 File) were estimated with linear mixed effects models from an energy balance, using collected data and an allometric approximation for resting metabolic rate with data from Krüger [58]. To allow this, parts of the experimental data first needed to be adapted. This included compensation for moisture uptake and faecal contamination of food when calculating ingestion rates, and application of moving averages to empirical data before analyses of trends in growth and ingestion rate (see Note SI6 in S1 File for details on data adaption). Data were analysed based on individual trends as well as average trends within each sex. Details on model parameter estimations from data can be found in Note SI7.1, SI7.2 in S1 File.

### 2.5 Model parameters estimated indirectly using optimization

For the growth model to be fully parameterized, three MGM parameters (*E*_*S*_, *γ*_*B*_, *a*_*N*_) remained to be estimated for males and four parameters (*E*_*S*_, *γ*_*BS*_, *γ*_*BR*_, *a*_*N*_) for females. These could not be obtained from literature, nor estimated directly from the experimental data. Instead, to obtain estimations of these parameters, an optimization procedure was performed where parameter values were adjusted to obtain least squares fit between predicted and empirical growth curves *W*(*t*). To avoid dependence of other parameters that were estimated with mixed effects models, the MGM growth equation as expressed by Eq (4) was not applied in this step. Instead, resting metabolic rate (*R*_*R*_) was decomposed into growth overhead costs (*R*_*G*_ = *E*_*S*_·*dW*/*dt*) and maintenance costs (*R*_*M*_); *R*_*R*_ = *R*_*G*_ + *R*_*M*_, enabling growth rate (*dW*/*dt*) to be expressed in terms of resting metabolic rate (approximated by a power allometry) and maintenance costs:

$$\frac{dW}{dt}=\frac{1}{E_{S}}[R_{R}-R_{M}],\;\mathrm{where}\;\{\begin{array}{l} \begin{array}{l} R_{R}\approx a_{R}W^{b_{R}}\\ R_{M,males}=\frac{\gamma_{B}W}{1-a_{N}W} \end{array}\\ \{\begin{array}{ll} R_{M,females}=\frac{\gamma_{BS}W}{1-a_{N}W} & ,W\leq W_{mat}\\ R_{M,females}=\frac{\gamma_{BS}W_{mat}}{1-a_{N}W_{mat}}+\frac{\gamma_{BR}(W-W_{mat})}{1-a_{N}(W-W_{mat})} & ,W\geq W_{mat} \end{array} \end{array}.$$ (5)

This growth equation is analogous to Eq (4), but is expressed in terms of resting metabolic rate parameters (*a*_*R*_, *b*_*R*_) instead of ingestion rate and feeding costs parameters (thus avoiding dependence of the latter two parameter groups that were estimated with mixed effects models, see Section 2.4). The parameters *a*_*R*_ and *b*_*R*_ were obtained from literature. To find initial values of unknown parameters (*E*_*S*_, *γ*_*B*_, *a*_*N*_ for males and *E*_*S*_, *γ*_*BS*_, *γ*_*BR*_, *a*_*N*_ for females), fixed effects were first estimated by an ‘inverse problem’ methodology [59, 60] that minimized least squares between model prediction and average experimental data using the numerical optimization function fmincon and ODE solver ode23s in Matlab. A non-linear mixed effects model (with random effects varying with individual) was then applied, combining the routines nlmefit and ode23s in Matlab. Optimization was performed over the whole growth interval at one time, with female growth rate described as a piecewise analytic function (with a break point at maturity). Main results from the parameter estimations are presented in SI9 Table in S1 File and details are provided in Note SI7.3 in S1 File. Implications of parameter estimations and model simplifications (section 2.2) are considered in Note SI9.1, SI9.2 in S1 File.

### 2.6 Model evaluation

#### 2.6.1 Evaluation of individual variation

Several model parameters were estimated from linear or non-linear mixed effects models, including random effects that vary with individuals. To evaluate the individual variation in parameter values, the standardized random effect (*SRE*) was calculated (in a similar way as the coefficient of variation) as the standard deviation (*σ*) of all individual random effects (*RE*), divided by the size of the fixed effect (*FE*):

SRE=σ(RE)/|FE|. (6)

For model parameters that were directly calculated from individual data, *SRE* simply is the coefficient of variation. A ‘good’ parameterization was assumed to yield low *SRE*s.

#### 2.6.2 Evaluation of model predictions

**Fig 3:**
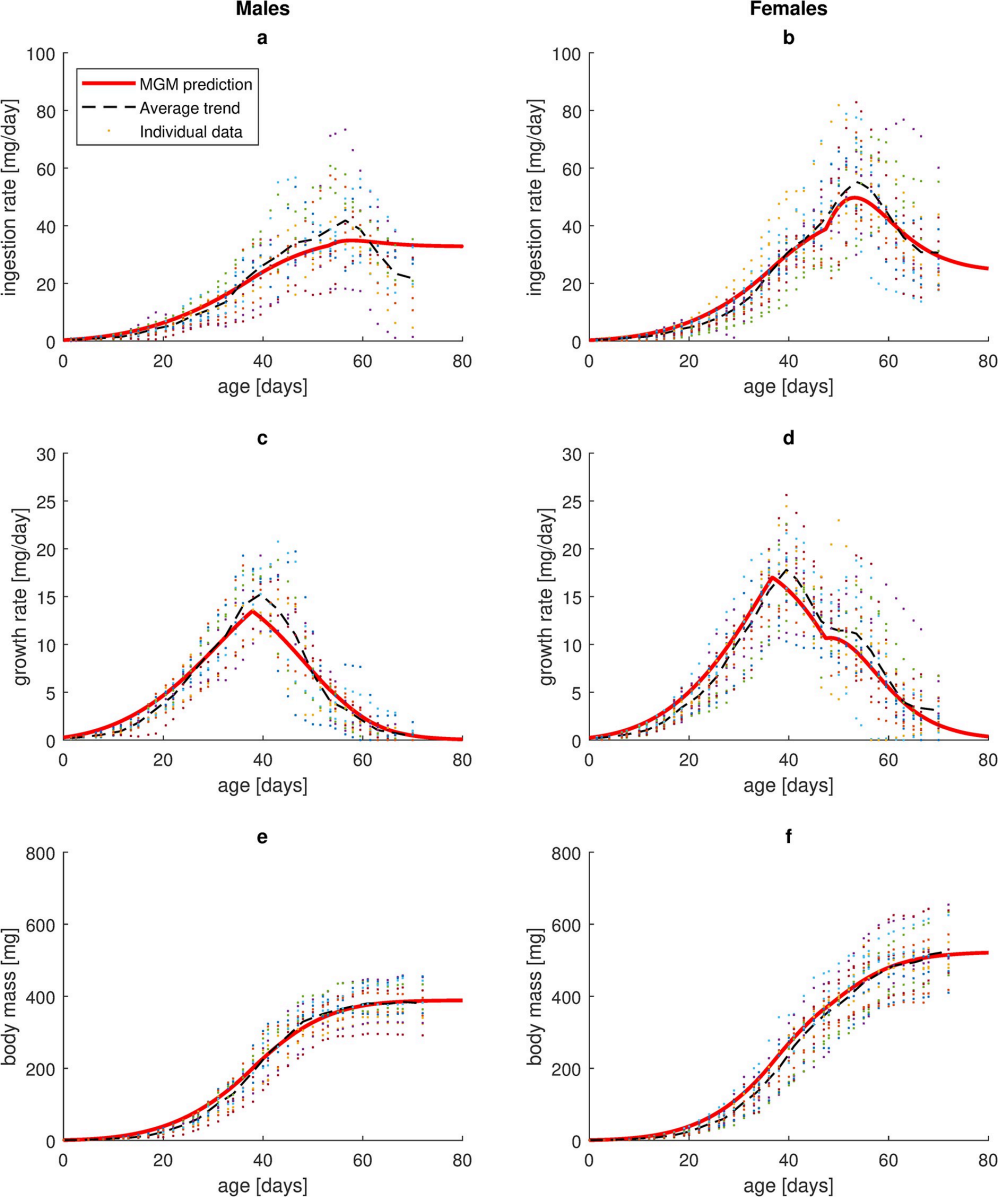
Observed and predicted ingestion rate, growth rate and body mass as functions of age. Data points represent individual empirical data for house crickets reared under ad libitum conditions at 28.6°C. Dashed curves are age-wise average values of individual empirical data. Model predictions (red curves) were obtained from numerical solutions to the growth equation (Eq (4)) with insertion of estimated fixed parameter values (SI9 Table in S1 File).

**Fig 4:**
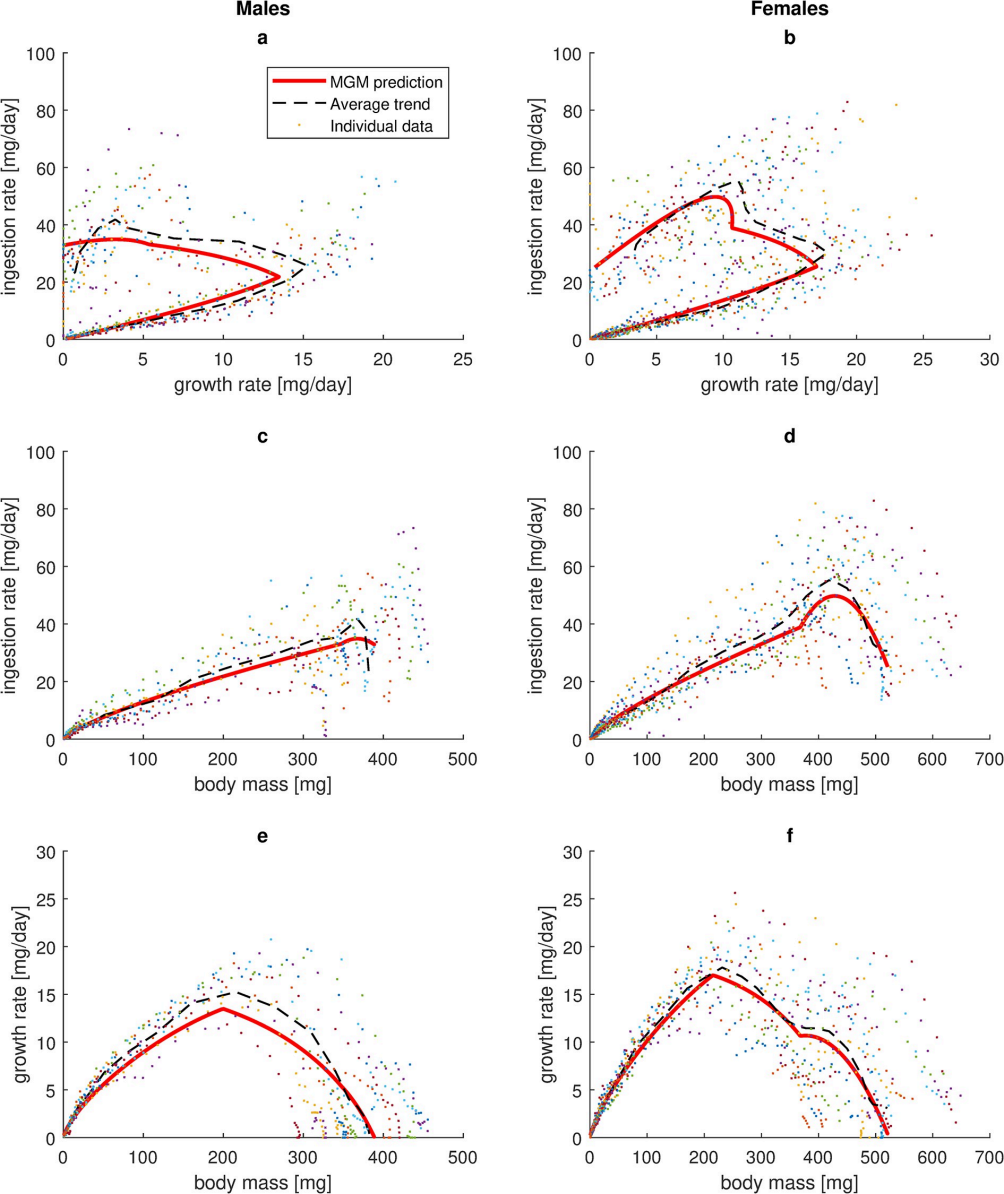
Observed and predicted ingestion and growth rate as functions of growth rate and body mass. Data points represent individual empirical data for house crickets reared under ad libitum conditions at 28.6°C. Dashed curves are age-wise average values of individual empirical data. Model predictions were obtained from numerical solutions to the growth equation (Eq (4)) with insertion of estimated fixed parameter values (SI9 Table in S1 File).

As an evaluation, estimated parameter values (SI9 Table in S1 File) were put into the MGM growth model (Eq (4)) and the differential equation was solved numerically with the Matlab routine ode23s. Predictions for averaged trajectories (across individuals) were obtained by using fixed effects of estimated parameter values, whereas predictions for each individual were obtained by including random effects. Average predicted curves for ingestion rate, growth rate and body mass were visually compared with empirical data (Figs 3 and 4). Goodness of fit (between predicted and empirical growth curves) was quantitatively evaluated by the measure:

$$GF=1-\frac{\sqrt{\sum_{i=1}^{n}{\left(O_{i}-E_{i}\right)}^{2}}}{\sqrt{\sum_{i=1}^{n}{\left(O_{i}-\bar{O}\right)}^{2}}}\;,\;\bar{O}=\frac{1}{n}\sum_{i=1}^{n}O_{i}.$$ (7)

Here *O*_*i*_ and *E*_*i*_ are observed and predicted value of data point *i* and *n* is the number of observations. Each data point represents a certain age for either one individual (individual trajectories) or the average of all male or female individuals (averaged trajectories). A perfect fit results in the maximum value *GF* = 1. The closer *GF* is to unity, the better the fit.

#### 2.6.3 Software

All data analyses and model predictions were performed with the numerical software MATLAB ^®^ (version R2021a, Mathworks Inc., Natick, MA, USA).

## 3. Results

### 3.1 Observed and predicted trends of ingestion and growth

Empirically collected data for house crickets were analysed for each sex separately. See Note SI6.6 and SI4 and SI5 Figs in S1 File for experimentally observed trends of ingestion rate, growth rate and body mass for individuals and the average male and female. Average-based ‘life-history parameters’, obtained directly from these data, are summarized in SI7 Table in S1 File.

Results of estimations of all model parameters, required to apply MGM for house crickets under ad libitum conditions, including fixed effects (and standardized random effects (*SRE*s) where applicable), are summarized in SI9 Table in S1 File (and commented in Note SI9.1 in S1 File).

The growth model (Eq (4)), based on estimated fixed parameter values (SI9 Table in S1 File), was able to produce visually good fits to empirical average data for ingestion rate and growth rate as a function of age (Fig 3A–3D, except a less good fit for late male ingestion rate (Fig 3A). The result is a predicted increase in body mass with age that well replicates the experimentally observed sigmoidal growth curves (Fig 3E and 3F), with a goodness of fit (Eq (7)) of 0.93 for males and 0.91 for females. When including random effects as well, the model reproduced empirical growth curves (body mass vs. age) for individuals with a goodness of fit of 0.72 for males and 0.61 for females.

Ingestion rate as a function of growth rate (Fig 4A and 4B) or body mass (Fig 4C and 4D), in MGM described by a power allometry until imago emergence and thereafter by a more complicated function of body mass, well captures the empirical trend for females, characterized by an ingestion rate that increases rapidly with size until imago emergence, shortly thereafter reaches a peak and then declines (Fig 4D). The fit was not as good for males, where individual variation was large and a general pattern less apparent (Fig 4C).

The distinctly hump-shaped curve for growth rate vs. body mass, observed in males, was captured by the model, although it tended to underestimate growth rate (Fig 4E). The initially similar curve, temporarily interrupted by a flat interval after imago emergence, observed in females, was well captured (Fig 4F). Sex-differentiated MGM formulations of maintenance costs (Eqs (SI25) & (SI27) in S1 File) were chosen to capture these patterns in the growth equation (Eq (4)).

### 3.2 Metabolic rates

**Fig 5:**
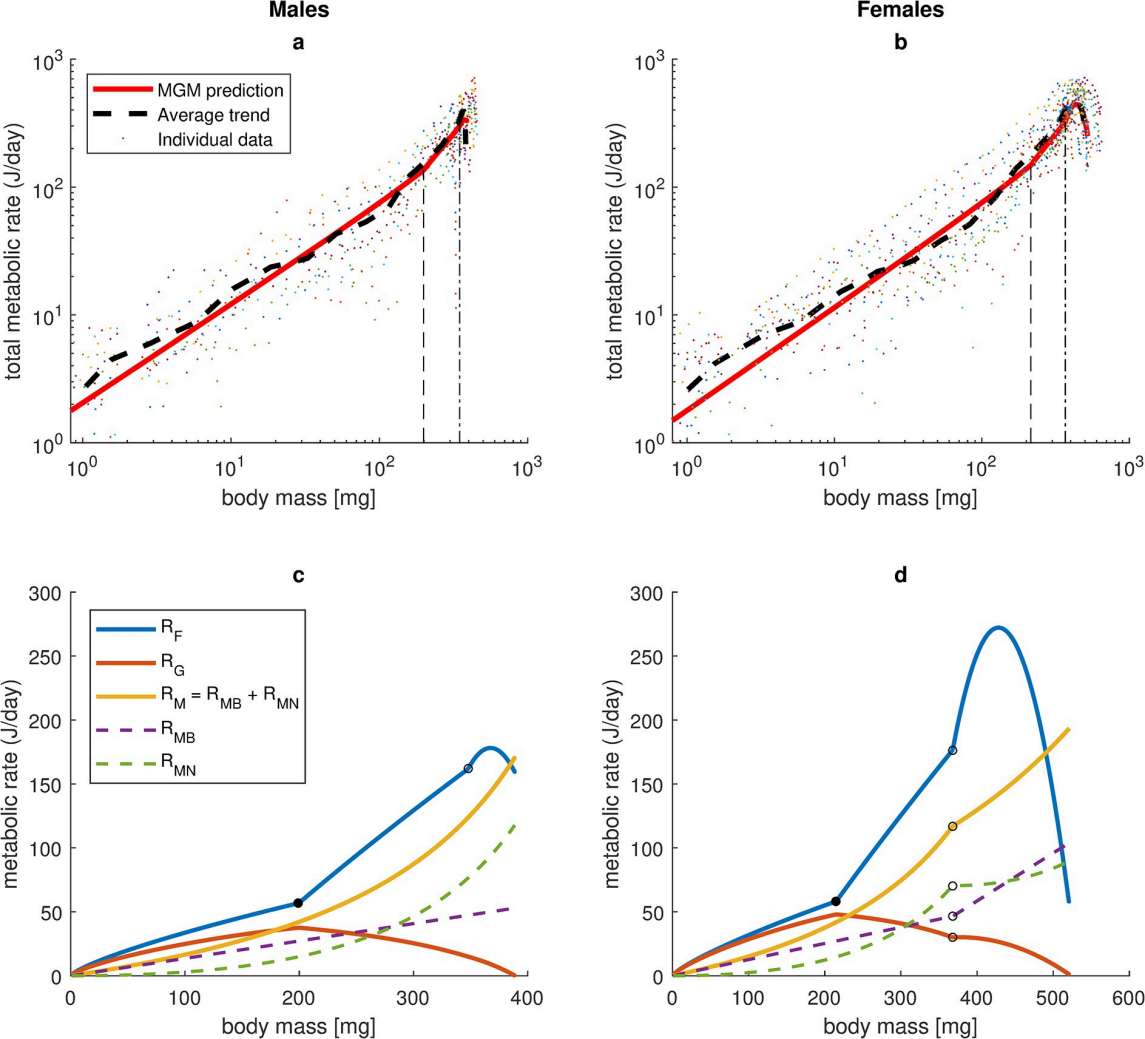
Observed and predicted metabolic rates. **a-b**) Total metabolic rate *R*_*tot*_ versus body mass (logarithmic scales) for male (**a**) and female (**b**) house crickets reared under ad libitum conditions at 28.6°C, predicted by MGM (Eq (SI5) in S1 File, solid curve) and calculated based on empirical data and literature estimates (Eq (SI37) in S1 File, dashed curve). Points represent calculated values for individuals. Dashed curves are average values of individual empirical data. Vertical dashed lines indicate mass at break point for feeding costs (*S* = *S*_1_). Vertical dash-dotted lines indicate mass at maturity. Predictions were obtained by inserting the numerical solution to the MGM growth equation (Eq (4) with estimated fixed parameter values from SI9 Table in S1 File) into model specifications of contributing metabolic components and summing (*R*_*tot*_ **=** *R*_*F*_ + *R*_*G*_ + *R*_*M*_). **c-d**) Predicted components of total metabolic rate versus body mass (linear scales) for males (**c**) and females (**d**). *R*_*F*_: Feeding costs (Eq (SI14) in S1 File). *R*_*G*_: Growth overhead costs (Eq (SI6) in S1 File). *R*_*M*_: Maintenance costs (Eqs (SI25) & (SI27) in S1 File). *R*_*MB*_: Basal maintenance costs (Eq (SI24) in S1 File). *R*_*MN*_: Negotiable maintenance costs (Eq (SI15) in S1 File). Filled circles (•) indicate break point for feeding costs (*S* = *S*_1_). Unfilled circles (o) indicate maturity.

The metabolic theory of ecology (MTE) assumes an allometric relation in the shape of a power law for total metabolic rate [1]. Here it was investigated if collected data and MGM predictions agree with this assumption. Though total metabolic rate was not directly measured in the experiment, it was indirectly calculated as the difference between assimilation and growth with insertion of collected experimental data for rates of ingestion and growth (Eq (SI37) and Note SI7.4 in S1 File) and was also predicted from the model as the sum of estimated MGM components (Eq (SI5) in S1 File). Both estimations resulted in average total metabolic rates that roughly follow power allometries (*R*_*tot*_ ≈ *a*_*T*_ ·*W*^*bT*^) during the juvenile phase, but around maturity the allometry (linear relationship on log-log scale) breaks (Fig 5A and 5B).

Based on indirect calculation of total metabolic rate (Eq (SI37) in S1 File) from experimental data (dashed curves in Figs 5A and 5B), the allometric exponents for the juvenile interval (*W* < *W*_*mat*_) were estimated to *b*_*T*_ = 0.783 ± 0.036 (males) and 0.772 ± 0.032 (females) from a linear mixed effects model (Note SI7.4 in S1 File). The allometric exponent *b*_*T*_ lacked individual variation (no random effects) and there was no significant sex difference. The normalisation constant *a*_*T*_, on the other hand, had large individual variation (*SRE*s for log_10_(*a*_*T*_) of 28–38%). This is in line with previous studies, indicating that allometric exponents of metabolic rates tend to be fixed within species, whereas normalisation constants show larger variation [17]. MGM based predictions of total metabolic rate (Eq (SI5) in S1 File), solid curves in Fig 5A and 5B) resulted in slightly higher allometric exponents; *b*_*T*_ = 0.836 (males) and 0.835 (females) for the entire juvenile interval (*W* ≤ *W*_*mat*_), and *b*_*T*_ = 0.789 (males) and 0.818 (females) for the shorter juvenile interval *S* ≤ *S*_1_ (before the slope of the feeding costs-ingestion rate relation turn steeper (Eq (SI36) in S1 File).

MGM provides detailed descriptions of all metabolic components that together constitute total metabolic rate (Eq (SI5) in S1 File, Fig 5C and 5D). During the juvenile phase, feeding costs (*R*_*F*_) increase with body mass (slowly for *S* ≤ *S*_1_ and faster for *S* ≥ *S*_1_). A second shift in the slope of *R*_*F*_ occurs at maturity (*W* = *W*_*mat*_) due to changes in ingestion rate (*S*), but *R*_*F*_ eventually drops (along with decreased *S*). Growth overhead costs (*R*_*G*_) follows the growth rate, peaking around *S* = *S*_1_. Basal maintenance costs (*R*_*MB*_) increase linearly with body mass, but with increased slope for mature females as a consequence of higher specific basal maintenance costs for reproductive tissue. Negotiable maintenance costs (*R*_*MN*_), and in turn total maintenance costs (*R*_*M*_), increase superlinearly with body mass due to increased relative allocation to negotiable costs. For females, *R*_*MN*_ shifts to a less steep slope at maturity since the increase in relative allocation to negotiable costs is switched from somatic to reproductive tissue. As a consequence of changed slopes in *R*_*MB*_ and *R*_*MN*_, also the slope of total maintenance costs (*R*_*M*_) shifts at maturity for females.

The *activity scope*, defined as the ratio between total (*R*_*tot*_ **=** *R*_*F*_ + *R*_*G*_ + *R*_*M*_) and resting (*R*_*R*_ = *R*_*G*_ + *R*_*M*_) metabolic rate, was calculated based on predicted metabolic components, yielding values in the interval 1.3 ≲ *R*_*tot*_/*R*_*R*_ ≲ 3. This agrees quite well with a general rule of thumb claiming that the average daily energy consumption for most organisms is roughly 2–3 times the resting metabolic rate [1], indicating that estimated metabolic components are plausible.

## 4. Discussion

A version of the Maintenance-Growth Model (MGM, Mauritsson and Jonsson [41]) was analysed for juvenile and adult growth under ad libitum conditions. This model captures a number of aspects that are not covered by common mechanistic growth models: 1) effects of changing relative allocation to negotiable maintenance costs during growth, reflecting a life-history dependent trade-off that potentially results in total maintenance costs that scale faster than linearly with body mass; 2) sex differences in allocation strategies; 3) explicit description of costs for finding and processing food; and 4) broken power allometry for ingestion rate at imago emergence. All this has consequences for allometric scaling of metabolic rates on which the metabolic theory of ecology [1] is based.

### 4.1 Growth model

MGM was empirically calibrated and evaluated for house crickets growing under ad libitum conditions. The most interesting findings of our analysis are:

First, by assuming increased relative allocation to negotiable maintenance costs during growth, the observed growth of an insect, from birth until terminated growth, could be well captured by MGM. Previously, it has been shown [41] that a growth equation of the general form (Eq (2) with *d* ≤ 1), representing all standard mechanistic growth models (AnaCat, DEB, OGM, Table 1), is not able to replicate growth trajectories until terminated growth in some insects, such as the ones observed here in house crickets (Fig 3E and 3F). For example, the high peak growth rate occurring at a relatively large body mass compared to ultimate size (Fig 4E and 4F) cannot be modelled, regardless of how model parameters are adjusted. It has been a common assumption that early ontogeny in insects is characterized by exponential growth, but data for a large set of species from various taxa suggest that insects generally grow slower than that during this phase [51]. As a result, common mechanistic growth models generally fail to accurately describe growth in insects also when applied to stages well below ultimate size, overpredicting growth rates during early ontogeny and underpredicting them at later stages [51]. In an effort to improve the description of growth in insects, Maino and Kearney [51] modified the standard DEB growth model (see Note SI9.4.2 in S1 File). By assuming increased assimilation efficiency with body size, this ‘insect DEB model’ succeeded in making good predictions for early ontogeny, but still could not satisfactorily describe late growth stages and in particular failed to accurately predict an asymptote. This was suggested to be explained by the existence of some developmental threshold, after which the proposed growth equation is no longer valid. However, though developmental thresholds may alter metabolic processes, energy conservation requires that growth (energy being stored in synthesised biomass) still balances assimilation minus respiration and this should be possible to express as a growth equation, as is done by MGM. When MGM was applied to house crickets, some threshold effects were indeed assumed (e.g. negotiable maintenance of reproductive tissue prioritised over somatic tissue in females after maturity). To summarize, MGM seems to be unique in its ability to capture insect growth from birth until ultimate body size. This relies on a superlinear relation between maintenance costs and body mass, which suggests that a linear relation, as modelled by OGM and standard DEB (Table 1), or a sublinear relation, as modelled by some versions of AnaCat (Table 1) and the insect DEB growth model [51], are incorrect for some (maybe most) insects.

Second, by modelling sex-differentiated strategies in allocation to negotiable maintenance costs after imago emergence, MGM was able to describe the qualitatively different growth trajectories, observed in males and females of an insect. Unlike males, adult females were assumed to prioritize growth and maintenance of reproductive tissue at the expense of somatic tissue. Gonadal growth is indeed known to be much higher in the female sex of house crickets [61], but this is common also in other species. Since females produce the largest gametes and generally invest more energy per offspring, they may be expected to prioritize growth and maintenance of reproductive tissue more than males.

Third, a power allometry for ad libitum ingestion rate was adequate throughout the juvenile phase for the observed species and may likely be so also for other insects, but at maturity the relationship broke and eventually ingestion rate decreased with further growth. This ‘broken allometry’ may reflect a hormonally regulated developmental threshold, which may be expected in determinate growers (like many insects), at which the adult stops feeding at its maximal capacity that is set by physical limitations (such as size of the gastrointestinal system). Among commonly applied mechanistic growth models, only DEB models apply allometric relations for feeding rates, but ‘broken allometries’ are not considered by the standard DEB model. The interpretation of observed allometric exponents for ingestion rate during the juvenile phase is further discussed in Note SI9.1 in S1 File.

Fourth, total feeding costs *R*_*F*_, including all energy expenses for finding and processing food, was assumed to be a monotonously increasing function of the ingestion rate *S*, but data suggested (Note SI7.2.2 in S1 File) that the function is more complicated than a simple proportional relation (SI10 Fig in S1 File) and was here described by a ‘hockey-stick’ where *R*_*F*_ initially increases slowly with *S*, but above a break point increases considerably faster with *S* (SI11 Fig in S1 File). This may reflect another developmental threshold (e.g. caused by a change in activity and/or foraging behaviour). In common mechanistic growth models (Table 1), foraging and food processing costs are not related to ingestion rates. In this analysis, the relation between feeding costs and ingestion rate was indirectly calculated from data by applying an energy balance. An alternative (but less likely) interpretation of the data is a sudden decrease in assimilation efficiency at the break point (which unless noted in Eq (SI35) in S1 File results in *R*_*F*_ increasing faster with *S*).

Fifth, growth overhead costs have been neglected in many previous growth models, only considering growth costs as energy bounded into newly synthesised biomass (e.g. early versions of OGM and the growth model by Makarieva et al. [62]. Consistent with previous findings [54, 63], this study indicates that growth overhead costs constitute a considerable part of total growth costs (*E*_*S*_/(*E*_*M*_ + *E*_*S*_) ≈ 31%, SI9 Table in S1 File) that should not be ignored in growth models.

## 4.2 Model complexity and number of parameters

In a hypothetical case where ingestion rate is allometrically related to body mass (*S* = *αW*^*β*^), feeding costs are proportional to ingestion rate (*R*_*F*_ *= k*_*F*_*S*) and maintenance costs are similar for all tissues (somatic or reproductive), the MGM growth equation (Eq (3)) at ad libitum is considerably simplified and by fusing model parameters together, a compact formulation that uses only four effective parameters (*a*, *β*, *c*, *a*_*N*_) is obtained:

$$\frac{dW}{dt}=\frac{1}{E_{M}+E_{S}}[eS-R_{F}-R_{M}]=aW^{\beta }-\frac{cW}{1-a_{N}W}\;,\;\{\begin{array}{l} a=(e-k_{F})\alpha /(E_{M}+E_{S})\\ c=\gamma_{B}/(E_{M}+E_{S}) \end{array}.$$ (8)

This formulation was applied in Mauritsson and Jonsson [41] to demonstrate that MGM was able to capture observed growth trajectories in some insects, where the Generalized Standard Growth Model (GSGM, Eq (2) with *d* ≤ 1) failed. Though this formulation of MGM may phenomenologically capture observed growth trajectories for male house crickets very well, the present study has shown that it is not mechanistically adequate. The constituent components of the MGM growth equation (*S*, *R*_*F*_ and *R*_*M*_) were analysed, using parameters that were estimated from literature or inferred from collected data by statistical models or ‘inverse model optimization’. It was found that ingestion rate (*S*), feeding costs (*R*_*F*_) and female maintenance costs (*R*_*M*_) required more complex descriptions than one might naively expect, resulting in a more complicated growth equation (Eq (4)). This formulation includes 13 (♂) or 14 (♀) model parameters (the parameters listed in SI9 Table in S1 File, except birth mass *W*_0_ that is an initial condition). However, by fusing parameters together, it is possible to give a description with 7 (♂) or 8 (♀) effective parameters. The model is thus more complicated than GSGM, that uses at most four (but usually less) effective parameters (see Table 1), but GSGM cannot accurately capture growth trajectories of the observed species [41]. The effective parameters have no direct biological interpretation, hence they cannot be estimated from literature nor calculated directly from observations, but they can be used as free parameters in an optimization procedure.

The large number of parameters introduced in MGM is a consequence of including many processes. We believe that ontogenetic growth is a result of several interactive and overlapping processes with different rates in different tissues at different phases during ontogeny and with significant variation among species, thus requiring many parameters to be captured by a model in specific cases. A ‘simpler’ model may seem ‘more general’ because it includes fewer, less species-specific parameters, but if it fails to provide a good fit for specific cases, a more complex model may be more useful and provide more insight despite the larger number of parameters.

In the analysis performed here, the pre-optimization parameter estimations imposed constraints on the model, resulting in 3 (♂) or 4 (♀) remaining free parameters for the optimization procedure, which was able to generate accurate fits to observed growth trajectories. As a comparison, GSGM could not do it with 3 (not even with 4) free parameters [41]. To analyse how total feeding costs (*R*_*F*_) depend on ingestion rate (*S*), feeding costs were calculated from an energy balance, using observed growth data and an allometric relation for resting metabolic rate based on data from literature [58]. Admittedly, a more direct measure of the resting metabolic would be preferable, but was not possible here. The analysis showed that *R*_*F*_(*S*) had the shape of a ‘hockey stick’; a piece-wise linear function with two slopes (*k*_*F*1_, *k*_*F*2_) and one break point (*S*_1_). To avoid dependence on parameter estimations from previous steps, the observed ingestion rate (not the parameterised description) and the previously applied allometry for resting metabolic rate (*R*_*R*_) were applied in the final parameter optimization procedure, using a formulation (Eq (5)) where feeding costs do not explicitly appear. Alternatively, the original growth equation (Eq (4)), where feeding costs appear explicitly, could have been used, either inserting estimated values of the feeding costs parameters (*k*_*F*1_, *k*_*F*2_, *S*_1_) or using them as free parameters. The latter alternative releases the constraint set by the *R*_*R*_ allometry, except qualitatively constraining *R*_*F*_(*S*) to a ‘hockey-stick’ relation, which is advantageous since *R*_*R*_ was not under control in the experiment. However, this increases the number of free parameters from 3 (♂) or 4 (♀) to 6 (♂) or 7 (♀), resulting in a much more difficult optimization procedure and expectation of better fits simply due to the increased number of degrees of freedom.

To conclude, the MGM growth equation in its most explicit formulation (Eq (4)), includes many model parameters, but this is not the reason why it captures observed growth trajectories better than GSGM. Furthermore, MGM is more general and flexible than other growth models and can be simplified to equally compact forms under certain circumstances, but reality is sometimes more complicated than that.

### 4.3 Estimation of model parameters

Not all parameters of MGM could be estimated directly from experiments in this study. Some were estimated prior to the experiments from the literature (the biomass energy density *E*_*M*_, the assimilation efficiency *e* and the allometric parameters for resting metabolic rate). The feeding costs parameters (*S*_1_, *k*_*F*1_, *k*_*F*2_) were indirectly inferred from an energy balance combined with empirical data and literature-estimated parameters. Others were indirectly estimated using an optimization procedure that minimised squared errors between predicted and observed growth curves; the specific growth overhead cost (*E*_*S*_), the mass-specific basal maintenance costs (*γ*_*B*_ (♂) or *γ*_*BS*_, *γ*_*BR*_ (♀)) and a parameter (*a*_*N*_) that affects the proportion of total maintenance costs that is allocated to negotiable parts. Admittedly, these methods adds uncertainty to the model calibration. Some parameters will be difficult to estimate in any direct manner, but we here propose some experiments that could be performed in future studies to obtain more direct estimates.

First, the total metabolic rate could be measured via oxygen consumption or CO_2_ production of growing individuals at regular time intervals in a similar experimental setup as the one adopted here. Second, the resting metabolic rate (of inactive non-feeding animals) could be measured for individuals that are deprived from food for some period (long enough to decimate feeding costs to negligible levels) before the measurement (instead of as here using existing literature estimates). These experiments would yield data series of total and resting metabolic rate versus age and body mass, which enables the estimation of feeding costs and related parameters in a way that does not rely on literature estimates. As follows from the energy balance of MGM, the feeding costs can be calculated as the difference between total and resting metabolic rate (*R*_*F*_ = *R*_*tot*_—*R*_*R*_). Third, the energy content per unit mass of provided food and collected faeces could be measured using a bomb calorimeter. In combination with direct measurements of ingestion and faecal production, this enables a direct measurement of assimilation (*A*), calculated as the difference between ingested energy and energy lost through egestion. This yields an empirical estimation of the assimilation efficiency *e* = *A*/*S* (as replacement for a literature estimate). Fourth, the biomass energy density *E*_*m*_ (energy content per unit body mass) could be measured for crickets of different size and sex, using a bomb calorimeter (instead of as here using existing literature estimates). With these experiments performed, the remaining parameters that require indirect estimation are those that were obtained from the optimization procedure (males: *E*_*S*_, *γ*_*B*_, *a*_*N*_, females: *E*_*S*_, *γ*_*BS*_, *γ*_*BR*_, *a*_*N*_).

Previous investigators [64] have suggested a way to estimate the specific growth overheads cost *E*_*S*_ from experiments where growth rate (*dW*/*dt*) and metabolic rate (*R*_*tot*_) are measured during early ontogeny. Under the assumption that maintenance costs (related to body mass) are negligibly small compared to growth costs during this stage, growth overheads (*R*_*G*_) were equated with metabolic rate (not considering feeding costs). The specific growth overhead cost can then be estimated as *E*_*S*_ = *R*_*G*_/(*dW*/*dt*) ≈ *R*_*tot*_/(*dW*/*dt*). A similar approach could be applied in a future study, accounting for feeding costs (*R*_*F*_), separately estimated with previously described method, resulting in *E*_*S*_ = *R*_*G*_/(*dW*/*dt*) ≈ (*R*_*tot*_ - *R*_*F*_)/(*dW*/*dt*). However, we do not think this would improve the estimation of *E*_*S*_ compared to the optimization method applied in our study. Our approach estimates *E*_*S*_ and a couple of parameters related to maintenance costs, such that predicted and observed growth curves agree well over the whole growth interval (from birth until terminated growth). The optimization method indirectly accounts for maintenance costs being small during early ontogeny (because they are related to body mass and data points for early ontogeny were included). In our study, the good agreement between predicted and observed growth rates during early ontogeny (and well beyond that, SI14a, SI14b Fig in S1 File), suggests that the specific growth overhead cost was well estimated (if estimated resting metabolic rate can be considered reliable).

In a previous study [41] and in the optimization procedure of this study (using a few free parameters related to maintenance and growth overheads) we found that accurate predictions of observed growth trajectories in an insect could only be obtained from the current growth model formulation (Eq (3)) if maintenance costs increased faster than linearly with body mass. We take this as quite strong support for the suggested pattern. However, there remain some model components related to maintenance that are truly difficult to differentiate based on empirical measurements. First, it is difficult to separate basal and negotiable maintenance costs. We simply assumed that basal maintenance costs scale linearly with body mass (the widely used standard assumption) and consequently, the negotiable maintenance costs must scale superlinearly with mass. An argument for this assumption is that the cell-specific cost for barely keeping the homeostatic balance within limits required for survival may roughly be constant for the average cell during growth, whereas priorities for negotiable costs may change. The whole body basal maintenance cost is then proportional to the total number of cells. If also the average cell size during growth can be considered constant, the basal maintenance cost is proportional to the body mass. Strictly, the latter assumption may often be violated [65], but the extent to which average cell size changes and thus how this simplification affects the results here remains to be studied. Second, if we accept that negotiable maintenance costs (*R*_*MN*_) increase faster than linearly with body mass (*W*), there may be several competing mathematical descriptions. We here assumed that the proportion of total maintenance costs that is allocated to negotiable maintenance costs increases linearly with body mass with parameter *a*_*N*_ as proportionality constant. Since other descriptions may be equally good, this may be considered merely as a phenomenological (not a mechanistic) description. Admittedly, it may be very difficult to separate basal and negotiable maintenance costs in any setup for empirical measurement. Speculatively, basal maintenance costs could be estimated in some experiment were the metabolic rate is measured for an organism that is kept at such scarce conditions that it barely survives. We leave it for future studies to tackle these challenges.

### 4.4 Life-history dependent trade-offs

MGM accounts for a life-history dependent trade-off between allocation to negotiable maintenance (keeping tissues in good shape) and growth (synthesis of somatic or reproductive tissue). It was found that increased relative allocation to negotiable maintenance during growth, resulting in a superlinear relation between total maintenance costs and body mass, could explain observed growth patterns in house crickets, but this is potentially applicable also to other insects where previous models have failed to explain data [51]. Suggestively, this makes biological sense by reflecting (*i*) a priority of growth at early life stages, (*ii*) increased priority of maintenance with increased amount of built-up capital (synthesised biomass), (*iii*) increased maintenance demands due to increased tissue complexity, (*iv*) increased costs of damage repair associated with ageing, or some combination of these. Increasing relative priority of maintenance over growth during ontogeny is supported by an energy allocation study of Daphnia [66]. Possibly, the hypermetric scaling of maintenance costs is the result of increased mass-specific costs of some specific negotiable maintenance functions. It remains to be studied which these might be. The available literature on regulation of maintenance in non-food-restricted, growing animals are mostly studies on immune function. Though negotiable maintenance costs include more than this, the associated energetic costs may be used as a proxy. Energetic costs for immune maintenance are known to be high, though difficult to measure [67, 68] and resistance against pathogens and other diseases is considered as an important life-history trait, that must be traded off against growth and reproduction [69, 70]. There is some empirical support for increased relative allocation to immune function with age in house crickets [71], but more empirical investigation is needed on size-dependent variation in allocation to maintenance of the immune system and other life-supporting functions during growth.

MGM models the life-history strategy of the observed species by applying positive allometric exponents (*b*_*NS*_, *b*_*NR*_) for relative allocation to negotiable maintenance of somatic and reproductive tissue (Eq (SI20) in S1 File). While some insects may increase their mass-specific maintenance during growth, other life-history strategies are thinkable and can be modelled by MGM. These include constant relative defence allocation (*b*_*NS*_ = *b*_*NR*_ = 0), resulting in maintenance costs being proportional to body mass (as assumed by many mechanistic growth models), and decreasing relative defence allocation (negative allometric exponents), resulting in a sublinear relation between maintenance costs and body mass (as predicted by some DEB growth models). Different allocation strategies may reflect the large variation in allometric exponents for total metabolic rate observed among animals and MGM is well prepared to model this.

### 4.5 Metabolic rates

Our estimations of total metabolic rate (Fig 5A and 5B) indicate that simple power laws may be acceptable approximations during ontogeny, but less so after maturity, consistent with other studies suggesting that multiphasic metabolic scaling is common [17, 27]. The allometric exponents for *total* metabolic rate *b*_*T*_ estimated here (0.783 for males and 0.772 for females) are somewhat lower than previous reports of the allometric exponent for *resting* metabolic rate in house crickets; *b*_*R*_ = 0.899 ± 0.050 [58] and 0.873 ± 0.273 [72]. There are (at least) two reasons for expecting differences. First, total metabolic rate was here not directly measured, but indirectly calculated as the difference between observed assimilation and growth (Eq (SI37) in S1 File) or predicted from MGM components (Eq (SI5) in S1 File). Both estimations are dependent on parameter values that were estimated from the literature (assimilation efficiency *e* and biomass energy density *E*_*m*_). Second, total metabolic rate includes feeding costs (costs for searching and processing food), not included in resting metabolic rate (measured for resting animals that have not recently been fed). Advocates of MTE and OGM [1, 49, 50] often assume that total and resting metabolic rate scale with body mass with the same allometric exponent. This assumption is probably too simplistic, since feeding costs (a large part of total metabolism) are closely linked to ingestion rate (as modelled by MGM), and ingestion rate may follow a different allometry than resting metabolic rate (that only includes maintenance and growth overhead costs), but see Jonsson [4] for a study where resting metabolic rate predicted the growth process in food-limited cohorts of active house crickets very well. In our study, feeding costs versus body mass had a steepened slope at a body mass of about 200 mg (Fig 5C and 5D), consistent with other studies suggesting that metabolism associated with activity and food processing tend to scale more steeply with body mass than resting metabolic rate [73–75].

## 5. Conclusions

Using experimental data, we have shown that a recently proposed growth model (MGM) that provides detailed descriptions of all components that constitute total metabolic rate, could well capture ontogenetic and post-mature growth until ultimate size of an insect species under ad libitum conditions. A number of tentative results were indirectly inferred from empirical data, analysed through the model: 1) most interestingly, total maintenance costs may increase faster than linearly with body mass; 2) costs for finding and processing food may depend on food intake in a more complicated way than a proportional relation; 3) differentiated allocation strategies between sexes may be essential for explaining their different growth trajectories; 4) growth overhead costs may constitute a considerable part of total growth costs, consistent with previous findings [54, 63]; and 5) power law descriptions of resting and total metabolic rate may be valid approximations during the ontogeny of determinate growers, but less so after maturity, consistent with the common finding of multiphasic metabolic scaling [27, 76, 77]. Also, it was observed that 6) power law descriptions of ingestion rates may only be applicable until maturity in determinate growers.

In summary, detailed examination of growth data for an insect species dismissed a simple growth model that uses only a few mechanistically meaningful parameters. The required model complexity of MGM resulted from non-simplistic patterns for ingestion rate, feeding costs and maintenance costs. Though a large number of model parameters were introduced, the performance of MGM does not rely on a large number of free parameters.

Though MGM was here analysed with data for only one hemimetabolous insect, it is believed to be a general model for individual growth and should be applicable also to other invertebrates and indeterminate growers. Instar-specific transitions in growth and metabolic scaling of holometabolous insects [17, 76, 77] may be modelled using stage-specific model parameters, including a threshold mass for the transition from larval to pupal stage.

Finally, MGM is well equipped to deal with growth under food limitation (as considered in a follow-up study [78]).

## Supporting information

S1 File (PDF)
