# Supplementary material for: A new mechanistic model for individual growth applied to insects under ad libitum conditions
Source: PLoS One. 2024 Sep 4;19(9):e0309664. doi: 10.1371/journal.pone.0309664 (PMC11373858; doi:10.1371/journal.pone.0309664)
Supplement: S1 File — (PDF) [file pone.0309664.s001.pdf]

SUPPORTING INFORMATION FOR

A New Mechanistic Model for Individual Growth Applied to Insects under Ad Libitum Conditions

Karl Mauritsson, Tomas Jonsson

This Supporting Information (SI) includes the following:

Note SI1. Major groups of mechanistic growth models.

Note SI2. General derivation of MGM.

- **Fig SI1**. Energy balance and energy flow in MGM.

Note SI3. Specification of MGM components for ad libitum conditions.

Note SI4. Detailed experimental setup.

- **Fig SI2**. Male and female house cricket.
- **Fig SI3**. Plastic box used in growth experiment.

Note SI5. Pre-experimental parameter estimations from literature.

- **Table SI1**. Scheme for calculation of food energy density.
- **Table SI2**. Growth and composition of tissue compartments of juvenile female house crickets.
- **Table SI3**. Growth and composition of tissue compartments of adult female house crickets.
- **Table SI4**. Scheme for calculation of assimilation efficiency of female house crickets.
- **Table SI5**. Obtained allometric parameters for resting metabolic rate in house crickets.

Note SI6. Management of experimental data and preparation for post-experiment parameter estimations.

- **Table SI6**. Parameters for food moisture uptake.
- **Fig SI4**. Experimentally observed ingestion rate, growth rate and body mass as functions of age for individuals and the average male and female.
- **Fig SI5**. Experimentally observed ingestion rate and growth rate as functions of growth rate and body mass for individuals and the average male and female.

Note SI7. Post-experiment empirical parameter estimations.

- **Table SI7**. Empirically estimated ‘life-history parameters’ for house cricket.
- **Table SI8**. Fixed effects of allometric parameters for total metabolic rate.
- **Fig SI6**. Linear mixed effects model for ingestion rate vs. body mass (1st interval).
- **Fig SI7**. Non-linear mixed effects model for ingestion rate vs. body mass (2nd interval).
- **Fig SI8**. Fixed effects model fits for ingestion rate vs. body mass.
- **Fig SI9**. Average metabolic costs versus body mass for male and female house crickets.
- **Fig SI10**. Average trends of feeding costs vs. ingestion rate for male and female house crickets.
- **Fig SI11**. Linear mixed effects model fit for feeding costs vs. ingestion rate for male and female house crickets.
- **Fig SI12**. Relative random effects of linear mixed effects model fit for feeding costs vs. ingestion rate.
- **Fig SI13**. Relative random effects for male and female model parameters obtained by non-linear mixed effects model fits between predicted and empirical growth curves.
- **Fig SI14**. Fixed effects solutions of body mass and growth rate, obtained from non-linear mixed effects model, and empirical data.
- **Fig SI15**. Individual solutions of body mass and growth rate, obtained from non-linear mixed effects model, and empirical data.
- **Fig SI16**. Linear mixed effects model applied to empirical data for $\log_{10}(R_{tot})$ versus $\log_{10}(W)$.

Note SI8. Results of model parameter estimations.

- **Table SI9**. Parameters for growth model of house cricket.

Note SI9. Supplementary discussion.

SI1. Major groups of mechanistic growth models

Many previous mechanistic models for growth under ad libitum conditions have the same general mathematical structure, expressing growth as the difference between two allometric terms (Eq. (2)). The first term represents some sort of contribution process, while the second term summarizes various types of consumption processes or costs. Three major groups of mechanistic growth models can be distinguished based on how these terms are interpreted and described (Table 1).

1) *Anabolism-Catabolism based* (AnaCat) *growth models* describe growth as the net result of anabolism (synthesis of new biomass) and catabolism (break-down of current biomass). One of the first mechanistic growth models [1] belongs to this category, describing growth as the net result of surface-related anabolism and volume-related catabolism. The famous von Bertalanffy (vB) growth model [2] describes anabolism as an allometric relation with flexible power exponent and catabolism as proportional to current body mass. It may be considered a quasi-mechanistic growth model since constants are treated as approximation parameters without physiological interpretation. The gill-oxygen limitation (GOL) model [3] is a minor modification of the Pütter model, describing anabolism as limited by the surface involved in oxygen uptake by water-living animals.

2) *Dynamic Energy Budget* (DEB) *growth models* [4-6] describe growth as the net result of assimilation and maintenance. Energy from ingestion (dependent on structural surface area and food availability) is assimilated into reserve (with some losses through excretion). A fixed fraction of energy mobilised from reserve is spent on somatic maintenance (proportional to the structural volume) and growth (increase of structural volume), whereas the rest is spent on maturation and maturity maintenance or reproduction and maturity maintenance. Anabolic and catabolic processes are indirectly accounted for in the sense that ingested food is assimilated into reserve, broken down via reserve mobilisation and converted into structure (growth). Heat losses (respiration) occur from maintenance processes and growth overhead costs. The growth equation is usually expressed in terms of structural volume (not body mass), but with constant food availability and constant energy density of reserves, body mass is proportional to structural volume.

3) *Ontogenetic Growth Models* (OGM) [7-13] describes growth as the net result of anabolism (metabolism) and maintenance, with metabolic rate described by a power law in accordance with MTE.

OGM has been updated and modified a number of times in order to deal with inconsistencies or include additional effects (temperature dependence and food limitation). See Note SI9.4.1 for a comparison between OGM and MGM.

SI2. General derivation of MGM

The Maintenance-Growth Model (MGM), developed by Mauritsson and Jonsson [14], describes ontogenetic and post-mature growth of a non-reproducing animal, based on an energy balance between energy assimilated from ingested food and expenses on growth and respiration (Fig SI1).

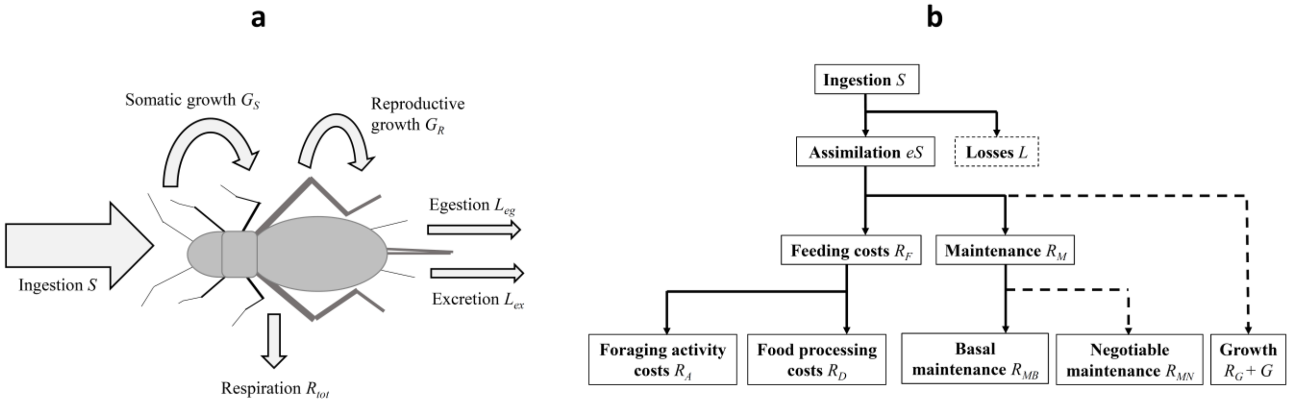

Fig SI1. a) Energy balance of a growing (non-reproducing) animal. Arrows represent fluxes of energy. *Ingestion S* is all energy that flows into the animal through consumed resources. *Respiration* R_{tot} is energy that fuels all metabolic processes, eventually released as heat to the environment. *Somatic growth* G_S is energy becoming bounded in synthesised somatic biomass. *Reproductive growth* G_R is energy becoming bounded in biomass of gonads, sperms and eggs. The transformation of energy from ingested food into usable forms include some energetic losses. *Egestion* L_{eg} is losses through faeces, whereas *excretion* L_{ex} is losses through urine and other excretes. **b**) Energy flow and allocation according to MGM. Boxes represent available energies or energetic costs. Solid arrows represent prioritized metabolic processes whose costs are paid first, while dashed arrows represent processes that can be down-regulated in response to what is available after prioritized costs have been paid. The total growth costs ($R_G + G$) include the energy becoming bounded in synthesized somatic and reproductive tissue ($G = G_S + G_R$) and the corresponding overhead costs ($R_G = R_{GS} + R_{GR}$).

The basic energy balance is expressed as:

$$S = R_{tot} + G + L \, . \qquad \text{(SI1)}$$

The ingestion rate S is measured as ingested energy per unit of time t and is assumed to be a function of the food supply S_{lim} and the body mass W (see Note SI3.3 for specification of ingestion rate in the version of MGM applied here):

$$S = S(S_{lim}, W) \, . \qquad \text{(SI2)}$$

Total growth ($G = G_S + G_R$) includes both somatic and reproductive growth, whereas total loss ($L = L_{eg} + L_{ex}$) includes losses through egestion and excretion (Fig SI1a). A fixed fraction (1 - e) of the ingested energy is assumed to be lost through egestion and excretion, where $0 < e < 1$ is the assimilation efficiency, mainly related to food type and quality. The energy balance can thus be expressed as a balance between assimilation rate (eS), total metabolic rate (R_{tot}) and growth (G):

$$eS = R_{tot} + G \, . \qquad \text{(SI3)}$$

The rate at which energy is bounded into synthesised biomass (G) is proportional to the rate at which the body mass increases:

$$G = E_M(W) \cdot \frac{dW}{dt} \, . \qquad \text{(SI4)}$$

Here $E_M(W)$ is the energy density of newly synthesised tissue (which depends on the relative proportions of carbohydrates, proteins and lipids at body mass W). It should be distinguished from the average energy density of the body, composed of tissues that have already been synthesised. However, there is no difference if the body composition is constant. See Note SI3.1 for specification of the energy density function $E_M(W)$ in the version of MGM applied here.

The total metabolic rate can be further divided into maintenance costs (R_M), feeding costs (R_F) and growth overhead costs (R_G):

$$R_{tot} = R_M + R_F + R_G \, . \qquad \text{(SI5)}$$

Growth overhead costs ($R_G = R_{GS} + R_{GR}$) include all indirect costs of somatic and reproductive growth, such as costs for assembling macromolecules from monomers, and is assumed to be proportional to the growth rate (dW/dt):

$$R_G = E_S(W) \cdot \frac{dW}{dt}. \qquad \text{(SI6)}$$

Here $E_S(W)$ is the specific growth overhead cost at body mass W (which may be differentiated between somatic and reproductive tissue as well as between somatic cell division and somatic cell growth, see Note SI3.2 for details/specification of the growth overhead costs function $E_S(W)$ in the version of MGM applied here).

Feeding costs (R_F) include all metabolic costs for searching and processing food and is assumed to be a monotonously increasing function of S (see Note SI3.4 for specification of the feeding costs function R_F in the version of MGM applied here):

$$R_F = R_F(S), \qquad \frac{dR_F}{dS} > 0. \qquad \text{(SI7)}$$

Maintenance R_M is energy spent on processes that keep the animal alive and in good shape, including maintenance of ion potentials across membranes, cell repair, immune activities and thermoregulation (in endotherms). Maintenance costs are assumed to depend on the relative food acquirement (φ), the body mass (W), body composition and the life history strategy of the animal (see Note SI3.5 for specification of the maintenance cost function R_M in the version of MGM applied here):

$$R_M = R_M(\varphi, W). \qquad \text{(SI8)}$$

The relative food acquirement φ is the ratio between realised ingestion rate S under current food regime and the ingestion rate S_{max} that is realised under ad libitum conditions at current body mass:

$$\varphi = S / S_{max}. \qquad \text{(SI9)}$$

Altogether, the relations above define how energy is acquired and utilized and they can be combined and rearranged into a general growth equation for ontogenetic and reproductive growth at ad libitum as

well as food limitation, describing how the growth rate dW/dt depends on ingestion rate (S), assimilation efficiency (e), maintenance costs (R_M) and feeding costs (R_F) in a way that is dependent on the life-history-determined trade-off between maintenance and growth-related costs ($R_G + G$):

$$\begin{aligned} eS &= R_{tot} + G = R_M(\varphi, W) + R_F(S) + E_S(W)\cdot\frac{dW}{dt} + E_M(W)\cdot\frac{dW}{dt} \Leftrightarrow \\ \frac{dW}{dt} &= \frac{1}{E_M(W)+E_S(W)}\left[eS(S_{lim}, W) - R_F(S) - R_M(\varphi, W)\right] \end{aligned} \quad . \qquad \text{(SI10)}$$

This is a general formulation of MGM. Components of Eq. (SI10) in the version of MGM applied here to ad libitum growth of house crickets are described in more detail below (Note SI3).

SI3. Specification of MGM components for ad libitum conditions

Below specific formulations are derived for the various components of MGM that will be used to calibrate and evaluate the model against empirical data collected from an experiment on house crickets (*Acheta domesticus*), growing under ad libitum conditions. Suggestions for more general formulations of MGM components, including food restriction, can be found in Mauritsson and Jonsson [14].

SI3.1. ENERGY CONTENT OF SYNTHESISED BODY TISSUE

Synthesised body tissue consists of somatic tissue (with energy density E_{MS}) and reproductive tissue (with energy density E_{MR}). MGM can describe changes in energy densities of somatic and reproductive tissues as a result of differences in relative proportions of synthesised carbohydrates, proteins and lipids (see Mauritsson and Jonsson [14]). Here, however, the parsimonious assumptions of non-changing fractions of lipids, carbohydrates and proteins during growth in somatic and reproductive tissue, and the same energy density for somatic and reproductive tissue ($E_{MS} = E_{MR} = E_M$, with E_M being a constant) are applied. The mass-specific energy content of fresh animal tissue is on average 7 J/mg [15], but shows variation across taxa and ontogenetic stages [8], here having been estimated to 6.1 and 6.3 J/mg for male and female house crickets respectively (Note SI5.2) from data collected by Woodring et al. [16].

SI3.2. GROWTH OVERHEAD COSTS

Growth overhead costs (R_G) are costs for synthesizing new biomass, which may be different for somatic and reproductive tissue as well as for somatic cell division and somatic cell enlargement. See Mauritsson and Jonsson [14] for specification of growth overhead costs in MGM that take these factors into account. Here, however, with assumptions resulting in constant biomass energy density E_M (see above), the consequential assumption of a single and constant specific growth overhead cost E_S is applied, required for predicting a continuous growth rate at imago emergence (from the growth equation), as indicated by empirical data (Fig SI4f, Fig SI5i).

SI3.3. INGESTION RATE

The ingestion rate S is measured as ingested energy per unit of time t. The maximum ingestion rate S_{max} is the ingestion rate that is realised under ad libitum conditions and is assumed to be allometrically related to body mass W, at least during a major part of ontogeny (up to a certain body size W'):

$$S_{\max} = \alpha W^{\beta}, \quad W \leq W'. \tag{SI11}$$

Here, β is an allometric exponent, α is a normalisation constant and W' is the body mass where the allometry potentially breaks. Allometries for ingestion rates are commonly assumed and empirically supported for many species [15]. The allometry may reflect size-dependent physical limitations of the gastrointestinal system as well as changes in foraging behaviour with increased body size. For indeterminate growers that grow throughout their whole lifespan (like many fishes), the allometry may very well hold generally. However, for determinate growers (like many insects), it may be the case that an animal under ad libitum conditions stops feeding at its maximal capacity at maturity or some time thereafter ($W = W'$). The animal may even reduce its ingestion rate below the achieved maximum level as it continues to grow. All of this could be the result of additional physiological processes kicking in at maturity.

The exact form of $S_{\max}$ for house crickets after maturity ($W > W' = W_{mat}$) was as a start unknown, but analyses of the experimental data (Fig SI5d-f) indicated that the rate of change of ingestion rate with body mass (dS/dW) is initially (for $W \leq W_{mat}$) inversely proportional to a power function of body mass,

but is after maturity also proportional to the difference between the body mass where the ingestion rate peaks and current body mass (see Note SI7.2.1).

Under food restriction, the ingestion rate S is limited by the food supply S_{lim} and the ad libitum ingestion rate S_{max} at current body mass W:

$$S = \min[S_{lim}, S_{max}(W)]. \tag{SI12}$$

SI3.4. FEEDING COSTS

Feeding costs include costs for searching and processing food. However, it may not be easy to separate costs for foraging activity R_A (necessary to acquire food) from digestive costs R_D (metabolic costs for digestion, assimilation, excretion and secretion). Since they presumably relate to ingestion rate in a similar way, it may be convenient to fuse them into a single metabolic component. Thus, feeding costs (R_F) include all metabolic costs for searching and processing food and is here assumed to be a monotonously increasing function of S:

$$R_F(S) = R_A(S) + R_D(S), \qquad \frac{dR_F}{dS} > 0. \tag{SI13}$$

The exact form of $R_F(S)$ is initially unknown, but a simple assumption is that feeding costs are proportional to the ingestion rate; $R_F = k_F \cdot S$. However, data for house crickets indicate that feeding costs increase faster with ingestion rate after the reach of some body size threshold (and ingestion rate is related to body size). This results in a more complex relationship, involving a break-point (at some ingestion rate S_1) that divides the description of feeding costs into two intervals:

$$R_F(S) = \begin{cases} k_{F1} \cdot S & , \quad S \le S_1 \\ k_{F1} \cdot S_1 + k_{F2} \cdot (S - S_1) & , \quad S \ge S_1 \end{cases}. \tag{SI14}$$

This formulation will be verified below for house crickets and values of the parameters S_1, k_{F1} and k_{F2} will be determined from the experimental data (Note SI7.2.2).

SI3.5. MAINTENANCE COSTS

It is assumed that maintenance costs are composed of two main parts: basal 'non-negotiable' maintenance costs (R_{MB}) that are a function of body size (and composition) and 'negotiable' maintenance costs (R_{MN}) that depend on food availability and life history strategy:

$$R_M = R_{MB} + R_{MN} \,. \tag{SI15}$$

If somatic and reproductive tissue (gonads, sperms, eggs, reproductive buffer) have different mass-specific basal maintenance costs (γ_{BS} and γ_{BR} respectively), the basal maintenance costs are separated into two parts:

$$R_{MB} = R_{MBS} + R_{MBR} = \gamma_{BS} W_S + \gamma_{BR} W_R \,. \tag{SI16}$$

Here, W_S and W_R are somatic and reproductive body mass, respectively.

'Negotiable' maintenance costs (R_{MN}) are all processes that can be 'tuned down' by the animal in order to save energy. They consist mainly of 'allocation to defence' (cell repair mechanisms, immune responses and maintenance of tissues that can be reduced in the short term without serious lethal danger) and 'negotiable' activity costs (non-necessary activity that is not directly linked to foraging, required for attaining the actual level of ingestion). As such, these 'negotiable' costs (R_{MN}) depend on (*i*) food availability and (*ii*) life history strategy. For a given body mass (W), the higher the level of food availability, the more energy will be available for 'negotiable' costs (R_{MN}) and growth-related costs (R_G + G) after basal maintenance costs (R_{MB}) and feeding costs (R_F) have been paid. How much of the available energy that is actually used for 'defence' (R_{MN}) and how much that is used for growth related costs (R_G + G) will be determined by the life history strategy (how defence of somatic and reproductive tissue is prioritized in relation to growth at different combinations of food availability and body size). The negotiable maintenance costs (R_{MN}) are here divided into somatic and reproductive parts:

$$R_{MN} = R_{MNS} + R_{MNR} \,. \tag{SI17}$$

The trade-off between energy allocated to defence and energy used for growth is here described by specifying negotiable maintenance of somatic tissue (R_{MNS}) as a fraction (ρ_S) of total somatic maintenance (R_{MS}), and negotiable maintenance of reproductive tissue (R_{MNR}) as a fraction (ρ_R) of total reproductive maintenance (R_{MR}). The fractions are functions of the relative food acquirement ($0 \leq \varphi \leq 1$) and tissue mass (W_S or W_R):

$$\begin{aligned} R_{MNS} &= \rho_S(\varphi, W_S) \cdot R_{MS} \quad , \quad 0 \leq \rho_S < 1 \\ R_{MNR} &= \rho_R(\varphi, W_R) \cdot R_{MR} \quad , \quad 0 \leq \rho_R < 1 \end{aligned} . \tag{SI18}$$

The fractions (ρ_S and ρ_R) are assumed to be regulated in response to the relative food acquirement by a factor $f(\varphi)$:

$$\begin{aligned} \rho_S(\varphi, W_S) &= f(\varphi) \cdot \rho_{NS}(W_S) \quad , \quad 0 \leq \rho_{NS} < 1 \\ \rho_R(\varphi, W_R) &= f(\varphi) \cdot \rho_{NR}(W_R) \quad , \quad 0 \leq \rho_{NR} < 1 \end{aligned} . \tag{SI19}$$

The defence regulation factor, $f(\varphi) \geq 0$, governs how negotiable maintenance costs are regulated in response to food restriction compared to ad libitum conditions. See Mauritsson and Jonsson [17] for a description of $f(\varphi)$ that may describe either downregulation ($f < 1$), no regulation ($f = 1$) or upregulation ($f > 1$) of negotiable maintenance under food restriction ($\varphi < 1$). Here, were ad libitum conditions ($\varphi = 1$) are considered, $f(\varphi) = f(1) = 1$.

The proportions of somatic and reproductive maintenance costs, ρ_{NS} and ρ_{NR}, that are allocated to negotiable parts under ad libitum conditions are assumed to relate to somatic and reproductive body mass by power functions:

$$\rho_{NS}(W_S) = a_{NS} W_S^{b_{NS}} \quad , \quad \rho_{NR}(W_R) = a_{NR} W_R^{b_{NR}} . \tag{SI20}$$

Positive allometric exponents (b_{NS}, $b_{NR} > 0$) corresponds to increasing relative defence allocation during growth, but constant ($b_{NS} = b_{NR} = 0$) or decreasing (b_{NS}, $b_{NR} < 0$) relative defence allocation is also thinkable.

Insertion of Eq. (SI18) into $R_{MS} = R_{MBS} + R_{MNS}$ and $R_{MR} = R_{MBR} + R_{MNR}$, yields negotiable maintenance costs expressed in terms of basal maintenance costs:

$$R_{MNS} = \frac{\rho_S}{1-\rho_S} \cdot R_{MBS} \quad , \quad R_{MNR} = \frac{\rho_R}{1-\rho_R} \cdot R_{MBR} \,. \tag{SI21}$$

Insertion of Eq. (SI21) into Eq. (SI17), with $R_{MBS} = \gamma_{BS} W_S$ and $R_{MBR} = \gamma_{BR} W_R$, yields total negotiable maintenance costs in terms of body mass components:

$$R_{MN} = \frac{\rho_S}{1-\rho_S} \cdot \gamma_{BS} W_S + \frac{\rho_R}{1-\rho_R} \cdot \gamma_{BR} W_R \,. \tag{SI22}$$

With Eqs. (SI19) and (SI20) inserted into Eq. (SI22), the total maintenance costs ($R_M = R_{MB} + R_{MN}$) at ad libitum ($\varphi = 1$) are obtained:

$$R_M(W) = \frac{\gamma_{BS} W_S}{1 - a_{NS} W_S^{b_{NS}}} + \frac{\gamma_{BR} W_R}{1 - a_{NR} W_R^{b_{NR}}} \quad , \quad \begin{cases} W_S = (1-p_R)W \\ W_R = p_R W \end{cases} \,. \tag{SI23}$$

Here, p_R is the proportion of body mass that is reproductive tissue. Due to differences in proportions of somatic and reproductive tissue, and in priority of their maintenance, the maintenance-growth trade-off may differ considerably between sexes of the same species. Notice that the denominators in Eq. (SI23) are considerably larger than zero as long as the basal maintenance costs (R_{MBS} and R_{MBR}) constitute considerable fractions of the total maintenance costs (R_{MS} and R_{MR}), which was fulfilled in this study and is generally expected.

MGM can describe sex differences in allocation to reproduction after maturity. This is relevant if females that have reached maturity prioritize gonadal growth at the expense of somatic maintenance, while mature males do not. To describe this, the specific somatic maintenance cost for **males** was assumed to be the same for somatic and reproductive tissue ($\gamma_{BS} = \gamma_{BR} = \gamma_B$). With γ_B constant, the basal maintenance cost (R_{MB}) is proportional to body mass (W):

$$R_{MB} = \gamma_B W \,. \tag{SI24}$$

Negotiable maintenance cost of male reproductive tissue is assumed to be negligible, formulated in the model by treating all growth as somatic, continuing also after maturity ($W_S = W$, $W_R = 0$). This is not entirely correct, but captures that male reproductive tissue is a small fraction of the total body mass and its maintenance is not prioritised over somatic tissue. To minimize the number of free model

parameters, a linearly increasing relative allocation to negotiable maintenance costs with body mass ($b_{NS} = b_{NR} = 1$) was assumed. Under ad libitum conditions ($\varphi = 1$), the total maintenance costs R_M for males are then obtained from Eq. (SI23) as:

$$R_M = \frac{\gamma_B W}{1 - a_N W} . \tag{SI25}$$

Notice that this formulation describes total maintenance costs as increasing faster than linearly with body mass. The standard assumption of a proportional relation ($R_M \propto W$) used by many previous growth models is obtained with $b_{NS} = b_{NR} = 0$, but this makes the model unable to capture observed growth patterns in house crickets (as demonstrated in Mauritsson and Jonsson [14]).

For **females**, the specific somatic maintenance cost is different for somatic and reproductive tissue. It was assumed that female growth until maturity is entirely somatic and then there is only reproductive (gonadal) growth. This is not entirely correct (see Tables SI2-3 and [16]), but captures the observation that females prioritise maintenance of reproductive tissue after maturity. The basal somatic maintenance then continues at a constant rate, while the basal reproductive maintenance rate is proportional to the amount of synthesised reproductive tissue:

$$R_{MB} = \begin{cases} \gamma_{BS} W & W \le W_{mat} \\ \gamma_{BS} W_{mat} + \gamma_{BR}(W - W_{mat}) & W \ge W_{mat} \end{cases} . \tag{SI26}$$

After maturity, the negotiable somatic maintenance cost continues at constant rate, while negotiable reproductive maintenance cost is an increasing fraction of the total reproductive maintenance cost. To minimize the number of free model parameters, it was assumed that $b_{NS} = b_{NR} = 1$ and $a_{NS} = a_{NR} = a_N$. Under ad libitum conditions ($\varphi = 1$), the total maintenance costs for females are then obtained from Eq. (SI23) as:

$$R_M = \begin{cases} \dfrac{\gamma_{BS} W}{1 - a_N W} & W \le W_{mat} \\ \dfrac{\gamma_{BS} W_{mat}}{1 - a_N W_{mat}} + \dfrac{\gamma_{BR}(W - W_{mat})}{1 - a_N (W - W_{mat})} & W \ge W_{mat} \end{cases} . \tag{SI27}$$

The proposed description of female maintenance enables MGM to account for the observed female-specific pattern of flattened growth rate for some period immediately after maturity, not observed in males (Fig SI5i). Gonadal growth is known to be much higher in female than in male house crickets as observed here [16], but this is common also in other species.

SI4. Detailed experimental setup

SI4.1. GROWTH EXPERIMENT

Eggs from house crickets (*Acheta domesticus*, Fig SI2) of a laboratory culture (initially purchased from a zoological supplier) were incubated under controlled conditions and 42 newly hatched nymphs were collected, weighted and reared individually in plastic boxes (195 x 195 x 110 mm) under ad libitum conditions for 72 days. Each box was equipped with a plastic dish for provision of food, a plastic dish filled with water and cotton, and a cardboard tube to provide shelter (Fig SI3). The lid was provided with small ventilation holes and coated with curtain fabric to prevent escapes. The surrounding temperature was held constant at T = (28.6 ± 0.9) °C for eggs and growing nymphs. A controlled amount of food (a mixture of finely grained commercial pellets for guinea pigs and rats, 50 % each) was provided at regular intervals two times a week (every third or fourth day), when the cricket was removed from the box and measured by weight. At each occasion, the shelter was brushed off from dry material and removed from the box together with food and water dishes. The cricket was then removed from the box and its body mass was measured on a scale with a precision of 0.1 mg. Young nymphs with a body mass less than 1.5 mg were weighted in groups of five individuals (in order to minimize measurement errors). Small individuals were captured with aspirators, while larger ones were captured with the cardboard tubes. All dry material (remaining food from previous feeding occasion and newly produced faeces) were collected from box and dishes, and weighted together on the scale. At some occasions, food and faeces were separated with the aid of tweezers and stereo microscope, to be measured separately on the scale (in order to collect data for estimation of relative egestion, see Note SI6.2). After finished measurements, a carefully controlled amount of new food (enough to avoid complete consumption) was provided to a cleaned food dish. The cotton was cleaned and fresh water provided to the water dish. Finally, shelter and cricket were replaced into the box. Age at maturity (entry of imago stage with fully developed wings) was recorded for each individual.

At the end of the experiment 39 surviving individuals remained (two dying in an early stage and one dying halfway through the study). Another 4 individuals were removed from the study, regarded as outliers (three individuals with very slow growth and one individual that never matured). Data for 35 individuals then remained, thereof 14 males and 21 females.

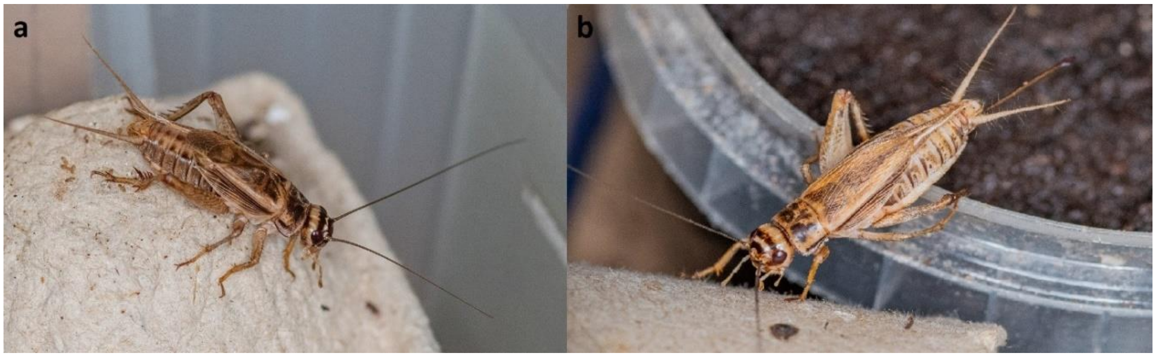

Fig SI2. Male (**a**) and female (**b**) house cricket (*Acheta domesticus*).

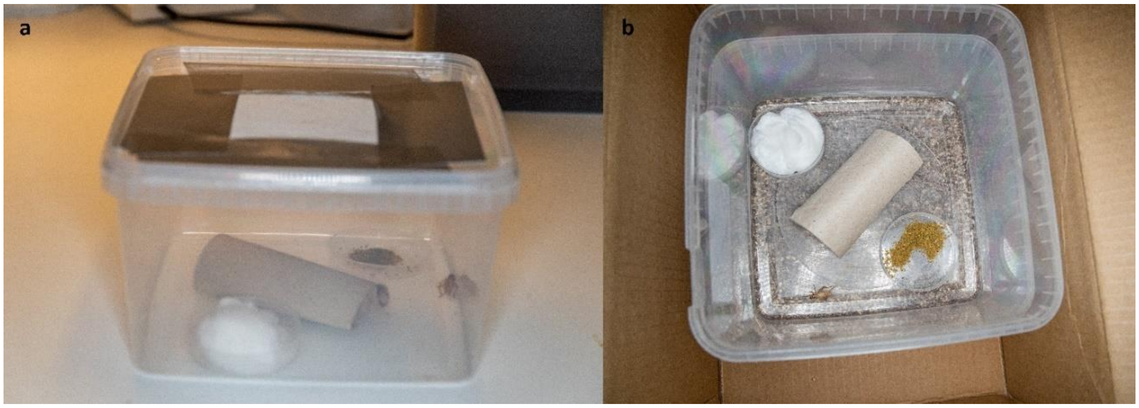

Fig SI3. Plastic box used in growth experiment, including food dish, water dish, shelter and cricket. (**a**) Side view of closed box. (**b**) Top view of open box.

SI4.2. FOOD MOISTURE MEASUREMENTS

The provided food was dry and finely grained, while the relative humidity within boxes was higher than in the surrounding, resulting in considerable moisture uptake and mass increase of provided food between measurement occasions. To accurately calculate food consumption from collected data, it was required to compensate for mass increase due to moisture uptake. A separate investigation was conducted to estimate the moisture uptake in food under current laboratory conditions. A total of 68

plastic boxes were equipped like boxes used in the growth experiment (except cricket), including food dish, water dish, cotton and cardboard tube. Various initial amounts of food (spanning from 10 to 600 mg) were weighted and then placed in the food dishes. Boxes were held under the same laboratory conditions as in the main experiment. The mass increase of the food (due to moisture uptake) was measured after 3 and 4 days (same time spans as used in the main experiment).

SI5. Pre-experimental parameter estimations from literature

A number of relevant parameters could be obtained prior to the experiment, estimated from the literature. These either included primary MGM parameters of Eq. (4) (energy density E_M and assimilation efficiency e), or 'associated parameters' (food energy density E_f, and resting metabolic rate normalisation constant a_R and allometric exponent b_R) from which other MGM parameters (E_S, γ_B, γ_{BS}, γ_{BR}, a_N) subsequently could be estimated when combined with experimental data.

The energy density (E_f) of provided food was calculated based on the nutritional content according to the commercial producers. The average energy density (E_M) of somatic and reproductive tissue in growing house crickets was estimated based on available data [16], accounting for sex differences in amount of reproductive tissue. The assimilation efficiency (e) of house crickets ingesting the provided food was estimated based on absorption efficiencies for different nutrients, measured by Woodring et al. [16]. To estimate resting metabolic rate (R_R) as a function of body mass (W), an allometric relation ($R_R = a_R \cdot W^{b_R}$) was applied with parameters (a_R, b_R) estimated based on available oxygen consumption data for inactive house crickets [18]. See Note SI5.1-SI5.4 below for details.

SI5.1. FOOD ENERGY DENSITY

The crickets were provided a mixture of 50 % guinea pig pellets and 50 % rat pellets. The energy density of the food was calculated based on the nutritional content according to the commercial producers and known energy densities of proteins, carbohydrates and fat (Table SI1), resulting in E_f = 13.4 J/mg.

Table SI1. Scheme for calculation of food energy density based on energy content of organic compounds and their fraction in and contribution to total energy content of provided food (mixture of 50 % guinea pig pellets and 50 % rat pellets). Energy contribution = Energy density × Fraction. Fractions are averages for both types of provided pellets (according to commercial producers). *Other: Crude fibre, crude ash, calcium, phosphorus, natrium.

**Constituent**	**Energy density** (J/mg)	**Fraction** (%)	**Energy contribution** (J/mg)
Proteins	17	13.5	2.3
Carbohydrates	17	55.9	9.5
Fat	37	4.5	1.6
Moisture	0	10	0
Other*	0	16.1	0
	**Total**	100	13.4

SI5.2. BIOMASS ENERGY DENSITIES

Amount and composition of the protein-rich residual body, the lipid-rich fat body and ovaries during the last nymphal stage and the first mature stage in female house crickets (*A. domesticus*) growing at 30 °C, have been reported by Woodring et al. [16]. These data were combined with known energy densities of carbohydrates, proteins and lipids to estimate the average energy density of synthesised somatic and reproductive tissue. During the first nymphal stages, all growth is somatic growth of a residual body. During the last nymphal stage, somatic growth is equally divided between continued residual body growth and growth of a fat body (Table SI2). The first mature stage (Table SI3) is dominated by gonadal growth with no net growth of somatic tissue (residual body growth is equated by consumed fat body). It was assumed that the composition of the residual body at the start of the last nymphal stage is representative of the average composition of the residual body during all nymphal stages. Data were converted from a dry mass basis to a wet mass basis, assuming that all biomass had a water content of 70%, the average water content in fresh cricket biomass [16].

Table SI2. Growth and composition of tissue compartments of juvenile female house crickets during the last nymphal stage [16]. Energy densities of compartments were calculated as the sum of energy contributions from different constituents (Energy density × Fraction).

**Compartment**	**Relative growth**	**Carbohydrates** [17 J/mg]	**Proteins** [17 J/mg]	**Lipids** [37 J/mg]	**Water** [0 J/mg]	**Other** [0 J/mg]	**Energy density** [J/mg]
Residual body	49 %	1 %	20 %	4 %	70 %	5 %	5.1
Fat body	51 %	2 %	9 %	20 %	70 %	0 %	9.0

Table SI3. Growth and composition of tissue compartments of adult female house crickets during the first mature stage [16]. Energy densities of compartments were calculated as the sum of energy contributions from different constituents (Energy density × Fraction).

**Compartment**	**Relative growth**	**Carbohydrates** [17 J/mg]	**Proteins** [17 J/mg]	**Lipids** [37 J/mg]	**Water** [0 J/mg]	**Other** [0 J/mg]	**Energy density** [J/mg]
Residual body	27 %	1 %	19 %	6 %	70 %	4 %	5.5
Fat body	-24 %	2 %	10 %	17 %	70 %	1 %	8.3
Ovaries	97 %	2 %	15 %	12 %	70 %	1 %	7.2

The energy density of somatic tissue during the first nymphal stages equals the energy density of the residual body and was obtained as $E_{MS1} \approx 5.1$ J/mg (Table SI2). The average energy density of somatic tissue during the last nymphal stage was estimated as a weighted average of compartment energy densities (with relative growth as weight), using data from Table SI2; $E_{MS2} \approx 7.1$ J/mg. The average energy density of somatic tissue during all nymphal stages was estimated as $E_{MS} \approx (E_{MS1}+E_{MS2})/2 \approx$ 6.1 J/mg and used as a general estimation of the energy density of somatic tissue for both males and females. The energy density of female reproductive tissue was estimated as a weighted average of compartment energy densities (with relative growth as weight), using data from Table SI3; $E_{MR} \approx 6.5$ J/mg. Males were assumed to consist entirely of somatic tissue, i.e. $E_M \approx E_{MS} \approx 6.1$ J/mg. The average biomass energy density for females accounts for both somatic and reproductive tissue; $E_M \approx (E_{MS}+E_{MR})/2 \approx 6.3$ J/mg.

SI5.3. ASSIMILATION EFFICIENCY

The assimilation efficiency of crickets ingesting provided food, was estimated based on published absorption efficiencies for different nutrients in female house crickets of the last nymphal and the first mature stage, reared under ad libitum conditions at 30 °C during two weeks [16], see Table SI4. Absorption efficiencies had been measured as fractions of nutrients absorbed, based on ingested and egested nutrient masses.

The assimilation efficiency e was calculated as $e = A_{tot}/E_f$, where A_{tot} is total assimilation (J/mg) and E_f is the food energy density (J/mg). The estimated assimilation efficiency was $e \approx 0.75$.

Table SI4. Scheme for calculation of assimilation efficiency of female house crickets, based on absorption efficiencies for different nutrients [16] and nutritional composition of provided food (according to commercial producer). Absorbed fraction = Absorption efficiency × Fraction, Assimilation = Energy density × Absorbed fraction.

Constituent	Energy density (J/mg)	Absorption efficiency	Fraction	Absorbed fraction	Assimilation (J/mg)
Proteins	17	81%	13.5%	11%	1.9
Fat	37	78%	4.5%	3%	1.3
Digestible carbohydrates	17	90%	45.4%	41%	6.9
Indigestible carbohydrates	17	0	10.5%	0	0
Other	0	0	26.3%	0	0
		**Total**	100%	55%	10.1

SI5.4. Resting metabolic rate parameters

To estimate resting metabolic rate (R_R) as a function of body mass (W) in our experiment, an allometric relation ($R_R = a_R \cdot W^{b_R}$) was applied, based on data of oxygen consumption in inactive house crickets (*A. domesticus*) reared under ad libitum conditions at 20 °C, collected by Krüger [18]. Linear regression was applied to Krüger's log-transformed data, yielding slope and intercept of the allometric relation ($\log_{10}(R_R)$ as a function of $\log_{10}(W)$). Oxygen consumption was converted to resting metabolic rate (using a translational factor) and metabolic rate at T_0 = 20 °C was converted to T = 28 °C by multiplication with the Boltzmann-Arrhenius factor (the temperature-dependent factor $e^{\varepsilon(T-T_0)/(\kappa T T_0)}$ in Eq. (1)), applying the activation energy ε = 0.65 eV/K and Boltzmann's constant $\kappa = 8.617 \cdot 10^{-5}$ eV/K [19]. As a comparison, allometric parameters for metabolic rate of inactive house crickets at 25 °C, presented by Hack [20], were converted to equivalents at 28 °C. Parameter values obtained from Krüger [18] and Hack [20] were similar, but confidence intervals from Krüger's data were more narrow for the allometric exponent (Table SI5).

Table SI5. Allometric parameters for resting metabolic rate in house crickets (mean ± 95 % CI). Estimations based on data from Krüger [18] were used to calibrate MGM parameters, whereas estimations from Hack [20] is presented as a comparison. Since confidence bounds are symmetric for $\log_{10}(a_R)$, they are asymmetric for the normalisation constant a_R.

**Data**	a_R [J/(mg·day^{b_R})]	b_R
Krüger [18]	0.728 [+0.568/-0.933]	0.899 ± 0.050
Hack [20]	0.710 [+0.229/-0.173]	0.873 ± 0.273

SI6. Management of experimental data and preparation for post-experiment parameter estimations

SI6.1. Moisture uptake in food

To compensate for food moisture uptake in calculations of food consumption, a power function was assumed for mass increase of food (due to moisture uptake) as a function of initial dry food mass W_{FD0}:

$$\Delta W_{FM} = a_M W_{FD0}^{b_M} . \tag{SI28}$$

The constants a_M and b_M were estimated by linear regression of logarithmic data; $\log_{10}(\Delta W_{FM}) = \log_{10}(a_M) + b_M \cdot \log_{10}(W_{FD0})$, resulting in a moisture uptake of 5-6 % (Table SI6), nearly independent of food mass since the relation between mass increase ΔW_{FM} and initial mass W_{FD0} was close to linear.

Table SI6. Parameters for food moisture uptake after 3 and 4 days of treatment (mean and 95 % CI).

	**3 days**	**4 days**
a_M	0.0525 [+0.0264/-0.0175]	0.0594 [+0.0320/-0.0208]
b_M	0.959 ± 0.083	0.909 ± 0.088

SI6.2. Relative egestion

To account for faecal mass in calculations of food consumption, where measurements of total remaining mass of food and faeces were used, it was assumed that the egestion W_E is a constant fraction (ξ) of the ingestion ΔW_F:

$$W_E = \xi \cdot \Delta W_F . \tag{SI29}$$

The relative egestion ξ was estimated from data, collected at occasions where remaining food and faeces were separated and measured separately. Ingestion ΔW_F (on a dry mass basis) was calculated as $\Delta W_F = W_{FD0} - W_{FD1}$, where W_{FD0} is the dry mass of provided food and W_{FD1} is the dry mass of remaining food. The latter was calculated based on measured wet mass of remaining food W_{FW1} with compensation for food moisture uptake by solving the equation $W_{FD1} + a_M W_{FD1}^{b_M} = W_{FW1}$. The equation has no analytical solution, but numerical solutions were obtained for each data point with the Matlab

routine *fzero*. A linear mixed effects model (W_E versus ΔW_F) was then applied to estimate ξ. However, random effects (accounting for individual variation) were negligible compared to the fixed effect. The fixed effect was $\xi = 0.65 \pm 0.02$ (mean and 95 % CI). As a comparison, relative egestion of house crickets in the study performed by Woodring et al. [16] can be calculated from data in Table SI4, yielding ξ = 1- total absorbed fraction = 1 - 0.55 = 0.45. However, the crickets in Woodring's study were reared under different conditions (different food and higher temperature) and faeces were dried before weighting, indicating that their estimate of ξ can be expected to differ from ours.

SI6.3. Ingestion rate

Ingestion rate was calculated based on the initial mass of provided food and the remaining total mass of food and faeces at next measurement, with compensation made for moisture uptake in food. The ingestion rate S (mg/day) at the middle of a time interval Δt (days), between food provision and measurement of remains, was calculated as the average over that interval; $S = \Delta W_F / \Delta t$. The ingestion ΔW_F (mg) is:

$$\Delta W_F = W_{FD0} - W_{FD1} . \qquad \text{(SI30)}$$

Here, W_{FD0} is the (known) dry mass of provided food and W_{FD1} is the (unknown) dry mass of remaining food. The faecal mass is $W_E = \xi \cdot \Delta W_F = \xi (W_{FD0} - W_{FD1})$, where ξ is the relative egestion. The wet mass of remaining food is $W_{FW1} = W_{FD1} + a_M W_{FD1}^{b_M}$, where a_M and b_M are food moisture uptake parameters (Table SI6). The measured (known) remaining mass of food and faecal can be expressed as $W_1 = W_{FW1} + W_E = W_{FD1} + a_M W_{FD1}^{b_M} + \xi (W_{FD0} - W_{FD1})$. The dry mass of remaining food W_{FD1} is obtained by solving the equation:

$$(1-\xi) W_{FD1} + a_M W_{FD1}^{b_M} = W_1 - \xi W_{FD0} . \qquad \text{(SI31)}$$

The equation has no analytical solution, but numerical solutions were obtained for each data point with the Matlab routine *fzero*. Once W_{FD1} has been obtained it can be inserted into Eq. (SI30) to yield the ingestion rate.

SI6.4. MOVING AVERAGE RATES

Observed rates of ingestion and growth at midpoints between two measurements were calculated as the observed ingestion or increase in body mass between the measurements, divided by the length of the time interval.

To smooth out short-term fluctuations in rates of ingestion and growth, moving averages were applied, based on three data points (evaluation point and adjacent time points). Each data point was weighted in proportion to its relative closeness to the evaluation point. More specifically, the moving average value of rate x_i at time point t_i was calculated as:

$$\bar{x}_i = \frac{1}{2(t_{i+1} - t_{i-1})}\left[(t_{i+1} - t_i)x_{i-1} + (t_{i+1} - t_{i-1})x_i + (t_i - t_{i-1})x_{i+1}\right]. \quad \text{(SI32)}$$

SI6.5. SEX-SPECIFIC AVERAGES

Sex-specific average values were calculated on an age-wise basis; for each measured age, the average of all individuals (of considered sex) was calculated.

SI6.6. EMPIRICAL TRENDS OF INGESTION AND GROWTH

Individual data and sex-specific averages of ingestion rate, growth rate and body mass as functions of age, growth rate and body mass are illustrated in Figs SI4-5.

Ingestion rate for both male (♂) and female (♀) house crickets increased rapidly with age until imago emergence, shortly thereafter reached a peak (around an age of 55 days) and then declined (Fig SI4c). Growth rate versus age followed a bell-shaped relationship for males, but the female curve flattened during a period after imago emergence, before decreasing rapidly again (Fig SI4f). For both sexes, average based growth rates peaked at an age of 40 days (⚥), significantly earlier than the peak in ingestion rate. The resulting growth curve was sigmoidal in both males and females (Fig SI4i) with imago emergence on average occurring on day 52 (♂) or 49 (♀). Increase of body mass after imago emergence was modest in males, but quite large in females (with much larger gonadal growth). As a result, females reached a considerable larger body mass (525 mg) than males (380 mg).

Ingestion rate followed a complex relationship with growth rate (Fig SI4c), but increased with body mass nearly in accordance with a power function until imago emergence, after which it continued to increase with body mass for another five days (Fig SI5f) and then decreased, along with decreased growth rate. The growth rate-body mass curve was distinctly hump-shaped for males, but the female curve flattened during an interval after imago emergence (Fig SI5i).

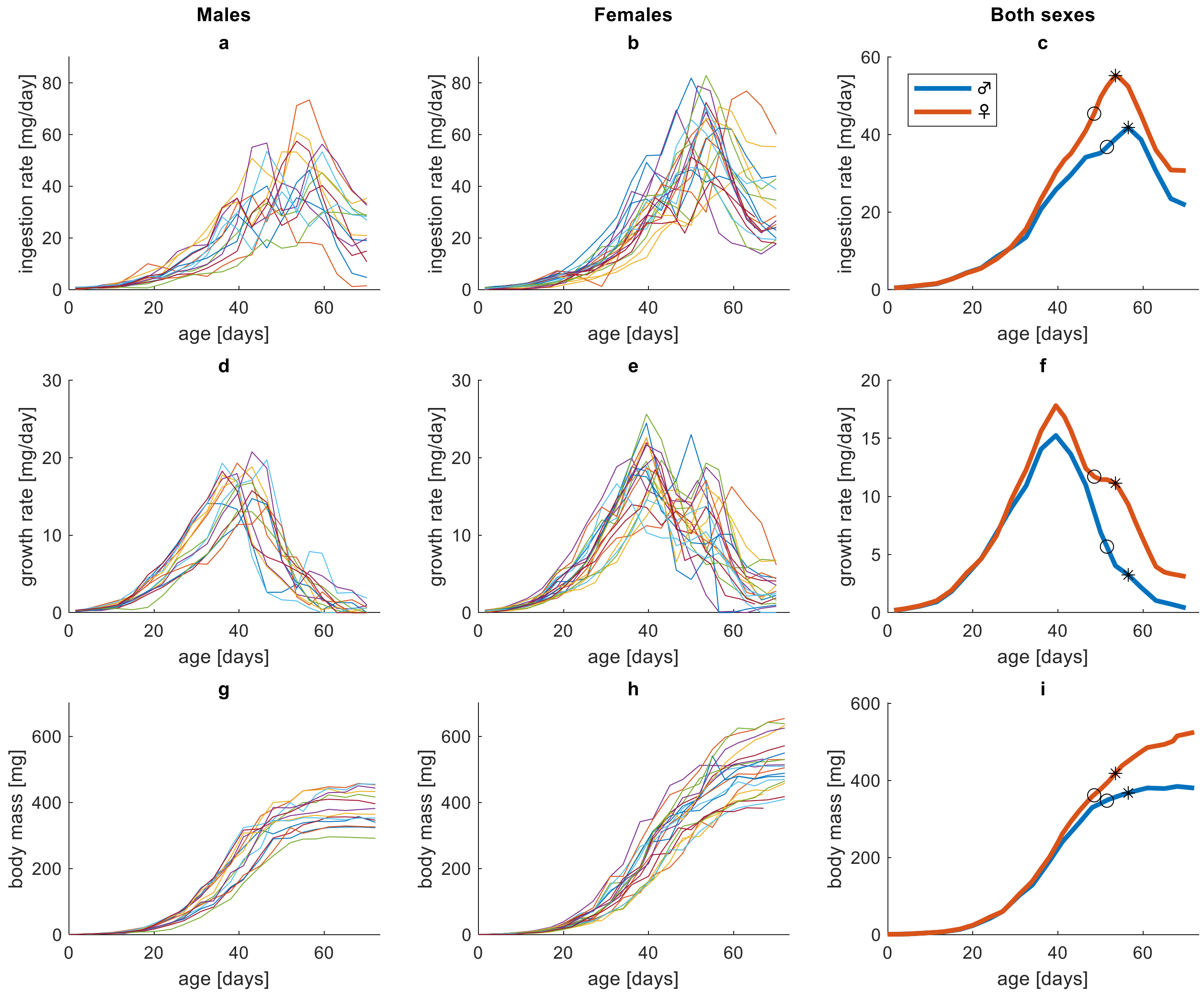

Fig SI4. Experimentally observed ingestion rate, growth rate and body mass as functions of age for individual males (left column), individual females (middle column) and the average male (♂) and female (♀) (right column) of house crickets reared under ad libitum conditions at 28.6 °C. First, moving averages were used to calculate rates of growth and ingestion for each individual. Then, average body mass, growth rate and ingestion rate were calculated for all individuals of each sex on an age-wise basis. Each curve in the left and middle column represents one individual. For the sex-specific average curves (right column), imago emergence (o) and peak of ingestion rate (∗) are indicated.

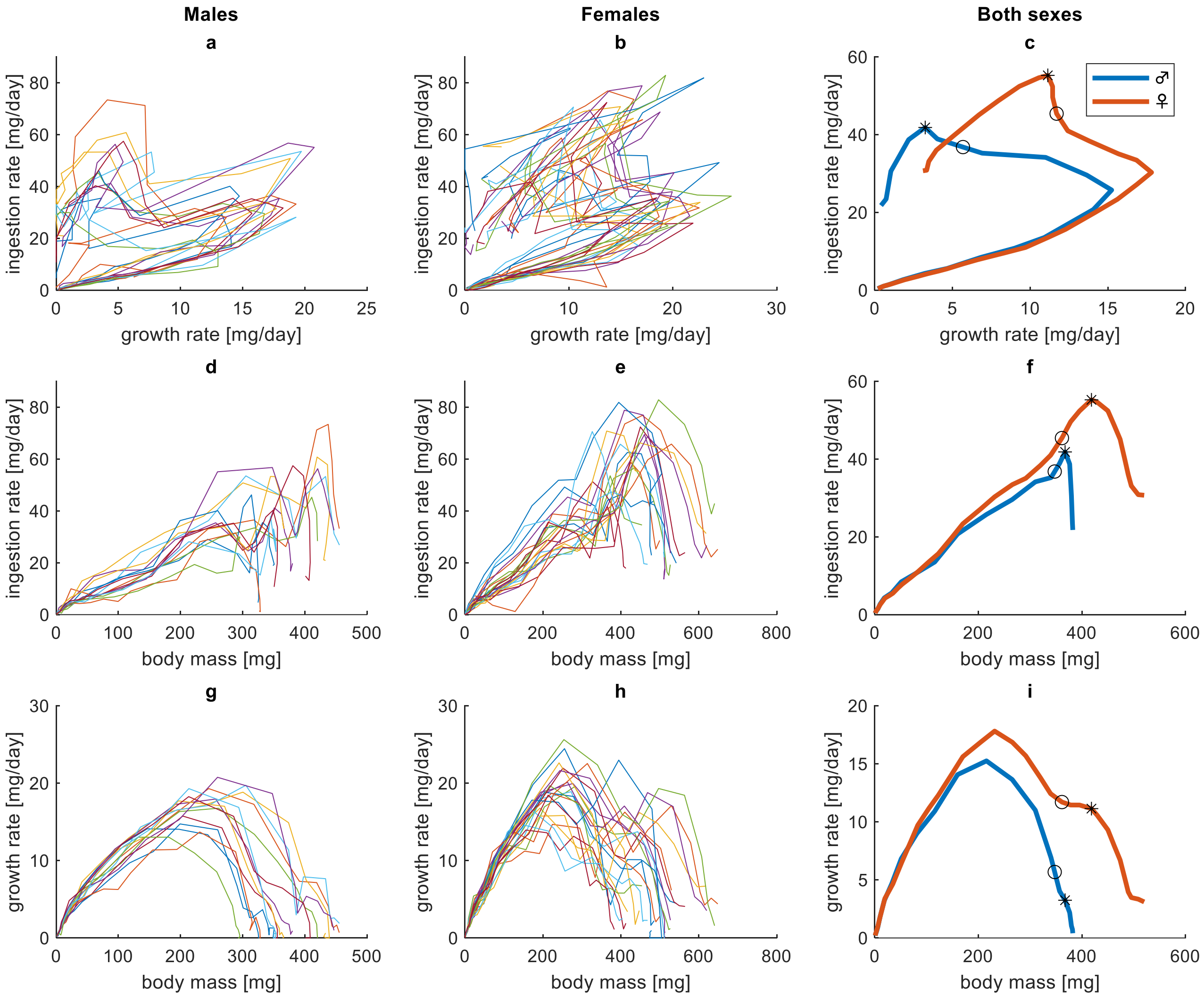

Fig SI5. Experimentally observed ingestion rate and growth rate as functions of growth rate and body mass for individual males (left column), individual females (middle column) and the average male (♂) and female (♀) (right column) of house crickets reared under ad libitum conditions at 28.6 °C. First, moving averages were used to calculate rates of growth and ingestion for each individual. Then, average body mass, growth rate and ingestion rate were calculated for all individuals of each sex on an age-wise basis. Each curve in the left and middle column represents one individual. For the sex-specific average curves (right column), imago emergence (o) and peak of ingestion rate (∗) are indicated.

SI7. Post-experiment empirical parameter estimations

SI7.1. PARAMETERS ESTIMATED DIRECTLY FROM EXPERIMENTAL DATA

A number of 'life-history parameters' were directly obtained from ingestion and growth measurements, by calculating averages for each sex separately. These are summarised in Table SI7.

Table SI7. Empirically estimated 'life-history parameters', obtained from data on age, body mass and ingestion of house crickets reared under ad libitum conditions at 28.6 °C. Mean values (± 95 % CI) were calculated for all individuals of each sex separately. For each individual, moving averages were applied to calculate rates of growth and ingestion.

			**Sex**	
**Parameter**	**Notation**	**Unit**	**Males**	**Females**
Birth mass	W_0	mg	0.8 ± 0.1	0.8 ± 0.1
Maximal growth rate	-	mg/day	15 ± 1	18 ± 1
Age at maximal growth rate	-	days	40 ± 2	40 ± 1
Mass at maximal growth rate	-	mg	216 ± 26	230 ± 20
Age at maturity	t_{mat}	days	52 ± 3	49 ± 2
Mass at maturity	W_{mat}	mg	348 ± 24	368 ± 19
Maximal ingestion rate	S^*	mg/day	42 ± 8	55 ± 6
Age at maximal ingestion rate	t^*	days	56 ± 2	55 ± 2
Mass at maximal ingestion rate	W^*	mg	367 ± 25	416 ± 24
Final mass (in experiment)	-	mg	380 ± 31	525 ± 36

SI7.2. PARAMETERS ESTIMATED FROM STATISTICAL MODELS OF EXPERIMENTAL DATA

SI7.2.1. Ingestion rate

According to the model, the ingestion rate under ad libitum conditions follows a power allometry, at least during the juvenile phase (Eq. (SI11)), reflecting size-dependent physical limitations and foraging behaviour. This seems to be an adequate description of growing house crickets. The observed ingestion rate (Fig SI5f), increasing with body mass until maturity, increasing further for a short period until peaking and then decreasing, may be the result of additional physiological processes kicking in at maturity (which can be expected for a determinate grower). To capture this, it was assumed that the rate of change of ingestion rate with body mass (dS/dW) is initially inversely proportional to an allometric power function (with exponent $1-\beta$), but after maturity dS/dW is also proportional to the difference between body mass at peak ingestion rate (W^*) and current body mass (W):

$$\frac{dS}{dW} = \begin{cases} \alpha\beta / W^{1-\beta} & , \quad W \le W_{mat} \\ k(W^* - W) / W^{1-\beta} & , \quad W \ge W_{mat} \end{cases} . \tag{SI33}$$

The formulation of the second interval ($W \geq W_{mat}$) is primarily suggested based on the empirical curves (Fig SI5f) and is qualitatively well supported (at least for females), but it can be argued that the formulation reflects some kind of mechanism. Until maturity ($W \leq W_{mat}$), the ingestion rate S increases with body mass W (being proportional to W^{β}), while the rate of increase (dS/dW) reduces (being inversely proportional to $W^{1-\beta}$). At maturity ($W = W_{mat}$), some physiological process kicks in. Suggestively, the ingestion rate is then adjusted to reach an ‘appropriate’ (optimal) ultimate body size. This can be achieved by a regulation mechanism where the rate of change (dS/dW) is still inversely proportional to $W^{1-\beta}$, but is also proportional to how far the animal is from the peak ingestion rate (W - W^*), resulting in an ingestion rate that increases with body mass until W^* is reached and then decreases.

Integration of Eq. (SI33) and continuity at $W = W_{mat}$ yields the ad libitum ingestion rate as:

$$S = S_{\max} = \begin{cases} \alpha W^{\beta} & , \quad W \le W_{mat} \\ \alpha W_{mat}^{\beta} + k\left[\dfrac{W^*(W^{\beta} - W_{mat}^{\beta})}{\beta} - \dfrac{W^{1+\beta} - W_{mat}^{1+\beta}}{1+\beta}\right] & , \quad W \ge W_{mat} \end{cases} . \tag{SI34}$$

For the first interval ($W \leq W_{mat}$), where Eq. (SI34) describes a linear relation in logarithmic space ($\log_{10}(S) = \log_{10}(\alpha) + \beta \cdot \log_{10}(W)$), a linear mixed effects model was applied to estimate the parameters (α, β) from logarithmized data, segregated by sex. Random effects (that vary by individual) were assumed for both slope and intercept. Other alternatives for inclusion of random effects were considered, but resulted in models with larger values of AIC (Akaike's Information Criterion), i.e. random effects on both the intercept $\log_{10}(\alpha)$ and the slope β explained the data better than alternative models.

For the second interval ($W \geq W_{mat}$), parameters were estimated differently for the sexes. For males, W^* was determined for each individual directly from the data and then averaged (observed body mass at maximum ingestion rate, Table SI7), and a non-linear mixed effects model (with random effects that vary by individual) was applied to estimate k from data (in linear space). For females, a more intricate data treatment was possible thanks to less individual variation and a more distinguished average trend. Hence, both k and W^* were used as free parameters with random effects when applying a non-linear mixed effects model to the female data (in linear space).

Results of statistical analyses, performed to estimate ingestion rate parameters (Eq. (SI34)), are presented in Figs SI6-7. Fixed effects and standardized random effects (*SRE*s, Eq. (6)) of obtained model parameters are presented in Table SI9. The fixed effects model fits (representing the average male and female) versus body mass over both analysed intervals are shown in Fig SI8.

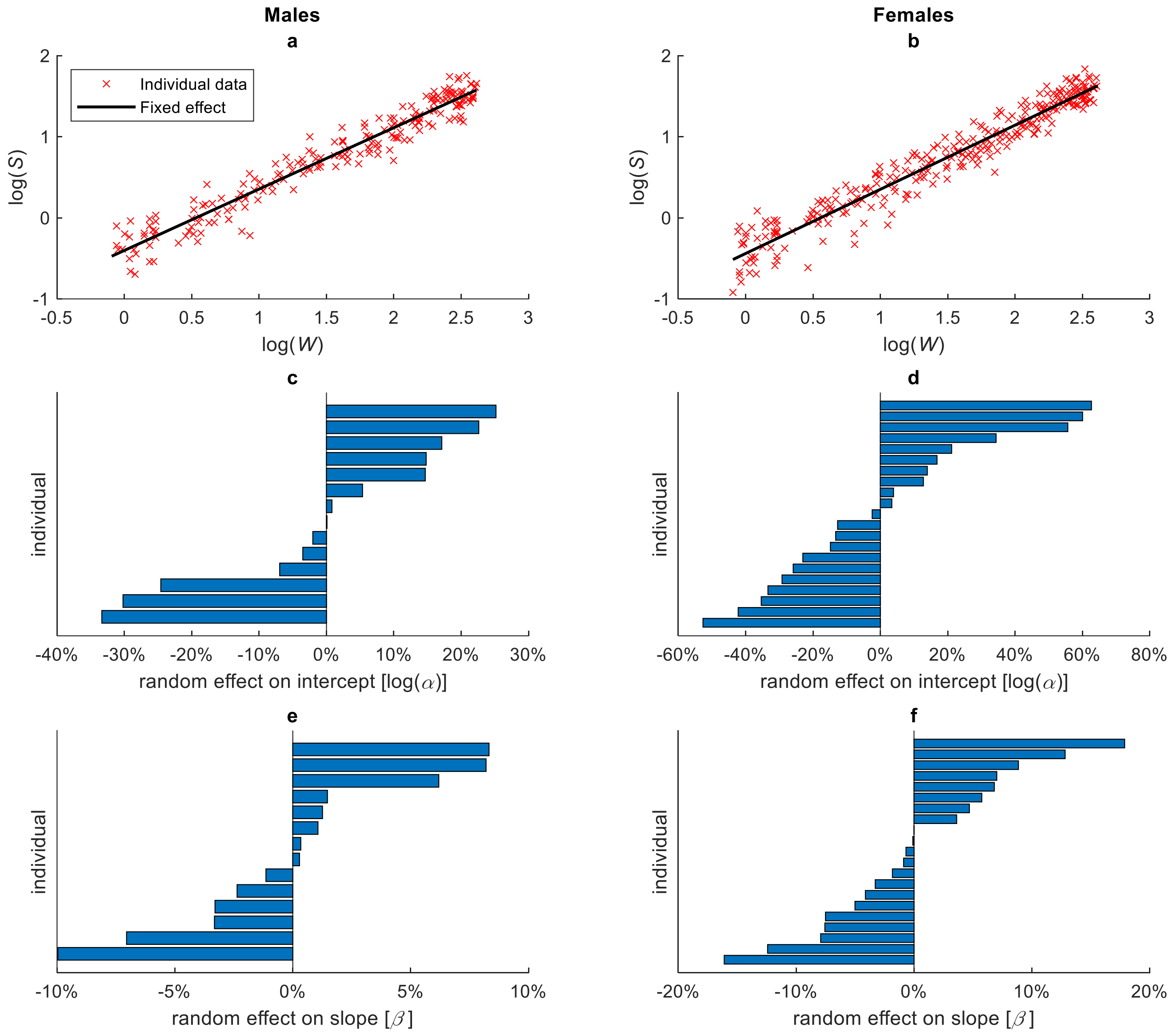

Fig SI6. Linear mixed effects model for ingestion rate vs. body mass of male (left column) and female (right column) house crickets reared under ad libitum conditions at 28.6 °C. The model was applied to empirical data for $\log_{10}(S)$ versus $\log_{10}(W)$ in the juvenile interval $W \leq W_{mat}$ (until imago emergence). An allometric relation $S = \alpha W^{\beta}$ was applied, where S is ingestion rate (mg/day), W is body mass (mg), α is the normalisation constant and β is the allometric exponent. Random effects (varying with individual) were included for both intercept and slope. Residuals were checked to be rather evenly distributed. Plotted relative random effects are individual random effects normalised by division with corresponding fixed effect.

Males: $\log_{10}(\alpha) = -0.402 \pm 0.059$ (*SRE*: 19 %), $\beta = 0.757 \pm 0.033$ (*SRE*: 5 %).

Females: $\log_{10}(\alpha) = -0.440 \pm 0.072$ (*SRE*: 34 %), $\beta = 0.791 \pm 0.035$ (*SRE*: 8 %).

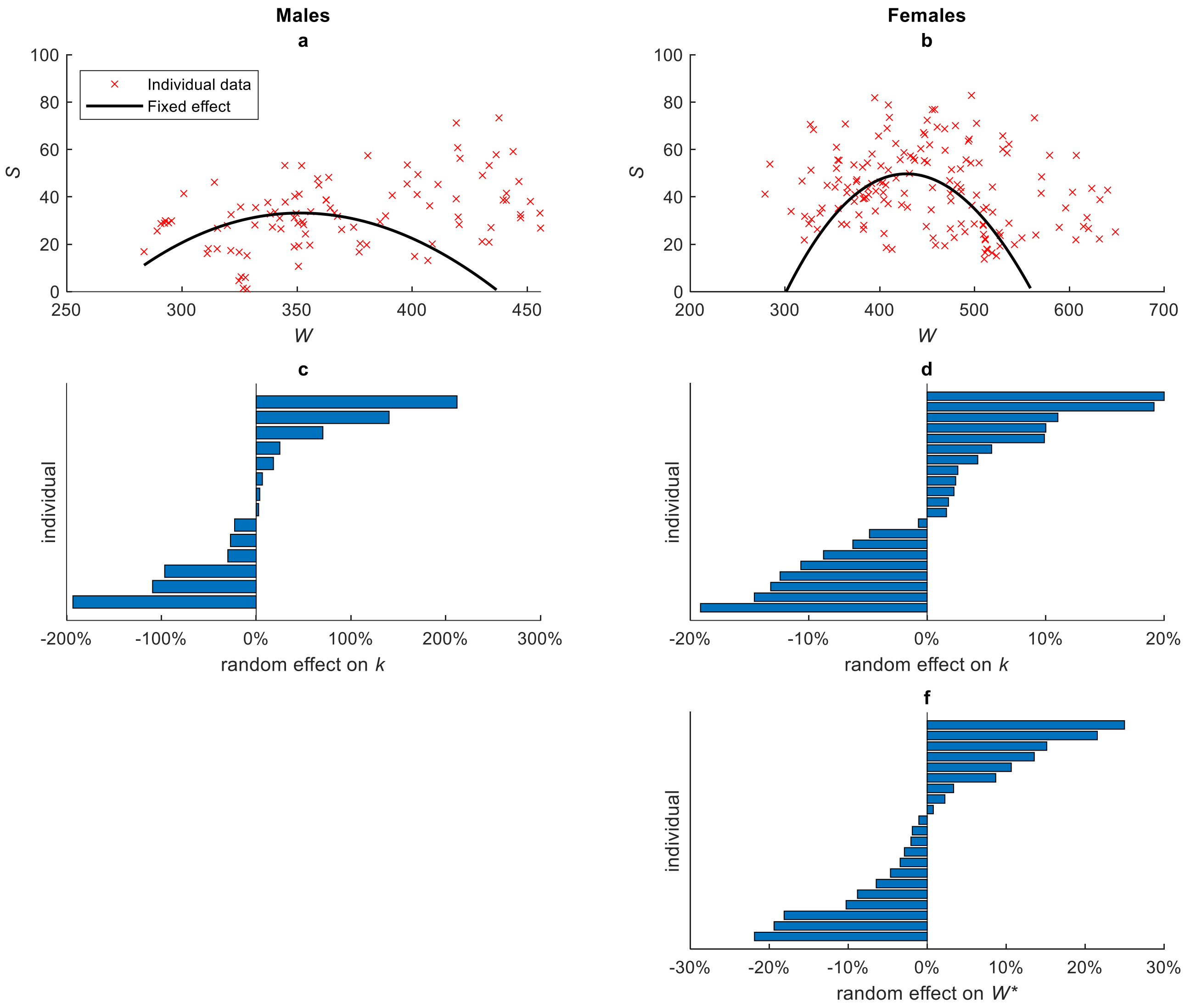

Fig SI7. Non-linear mixed effects model applied to empirical data for ingestion rate *S* versus body mass *W* in the post-mature interval ($W \geq W_{mat}$) of male (left column) and female (right column) house crickets reared under ad libitum conditions at 28.6 °C. Plotted relative random effects are individual random effects normalised by division with corresponding fixed effect. Males: k = 0.039 ± 0.050 mg/(mg$^{1+\beta}$·day) (*SRE*: 100 %). Females: k = 0.021 ± 0.005 mg/(mg$^{1+\beta}$·day) (*SRE*: 11 %), W^* = 428 ± 23 mg (*SRE*: 13 %).

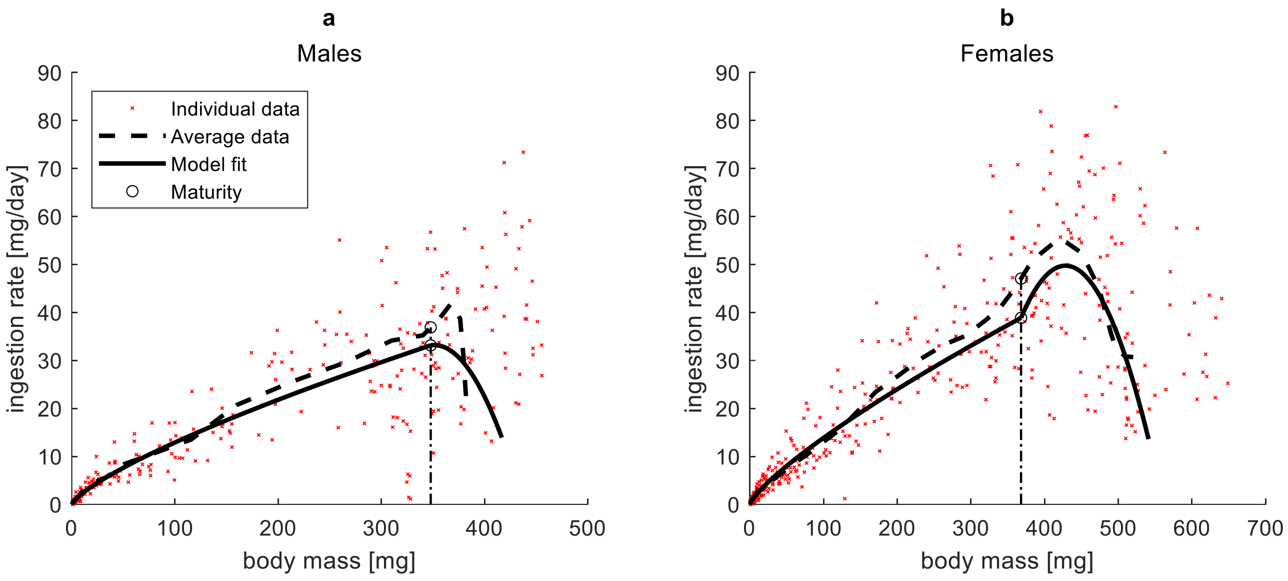

Fig SI8. Fixed effects model fits for (**a**) male and (**b**) female ingestion rate versus body mass, over both analysed intervals ($W \leq W_{mat}$ and $W \geq W_{mat}$) for house crickets reared under ad libitum conditions at 28.6 °C. Data points (x) represent individual empirical data. Dashed curves are average empirical trends. Vertical dash-dotted lines indicate maturity W_{mat}.

SI7.2.2. Feeding costs

Feeding costs include costs for foraging activities and metabolic costs of food processing. To analyse the relation between feeding costs R_F and ingestion rate S, feeding costs were indirectly estimated from collected data, based on the energy balance and estimations of energetic components. Assimilation rate ($A = eS$) equals the sum of total metabolic rate (R_{tot}) and biomass synthesis ($G = E_M \cdot dW/dt$), where R_{tot} may be further decomposed into feeding costs (R_F) and resting metabolic rate (R_R), assuming that the latter does not include any costs for food processing; $A = R_{tot} + G = R_F + R_R + G$. If resting metabolic rate is described by an allometric relation ($R_R = a_R W^{b_R}$), feeding costs can be expressed as:

$$R_F = A - R_R - G = eS - a_R W^{b_R} - E_M \frac{dW}{dt} . \quad \text{(SI35)}$$

Resting metabolic rate parameters (a_R and b_R) estimated from data presented by Krüger [18], and experimental data for ingestion rate S and growth rate dW/dt for different body masses, were applied to calculate feeding costs for each individual of the study. Obtained average values of metabolic components versus body mass are shown in Fig SI9. Sex-specific average feeding costs versus ingestion rate are presented in Fig SI10.

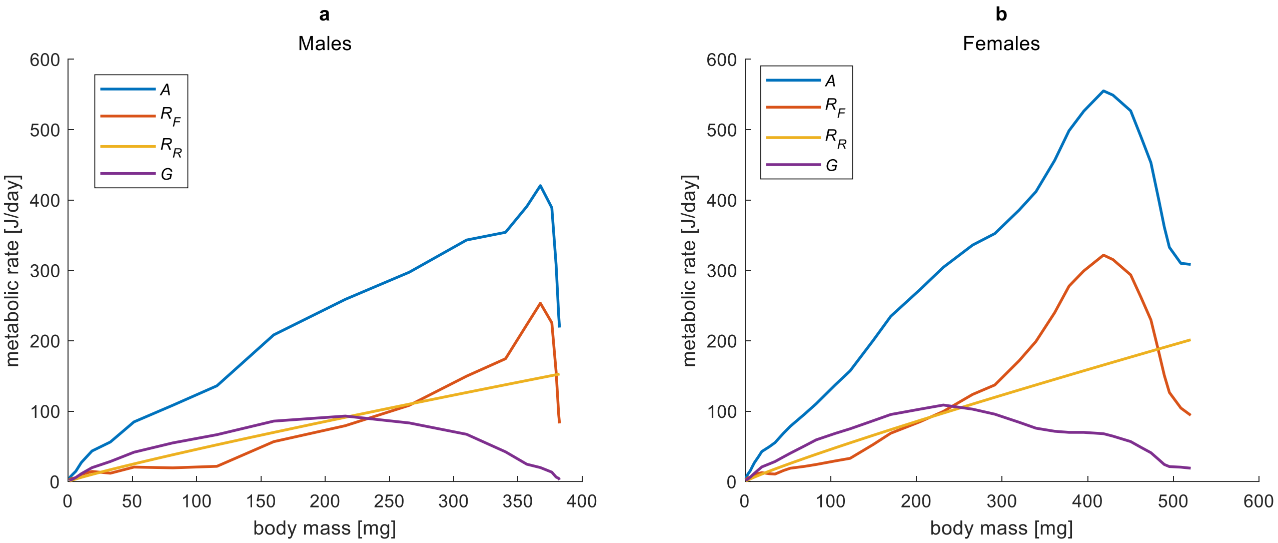

Fig SI9. Average metabolic components versus body mass for (**a**) male and (**b**) female house crickets reared under ad libitum conditions at 28.6 °C. A: Assimilation rate. R_F: Feeding costs. R_R: Resting metabolic rate. G: Biomass synthesis. Assimilation equates the sum of all metabolic components and growth ($A = R_F + R_R + G$). Assimilation rate and biomass synthesis were estimated from experimental data while resting metabolic rate was estimated from allometric relationship based on data in Krüger [18]. Feeding costs were then calculated using Eq. (SI35)).

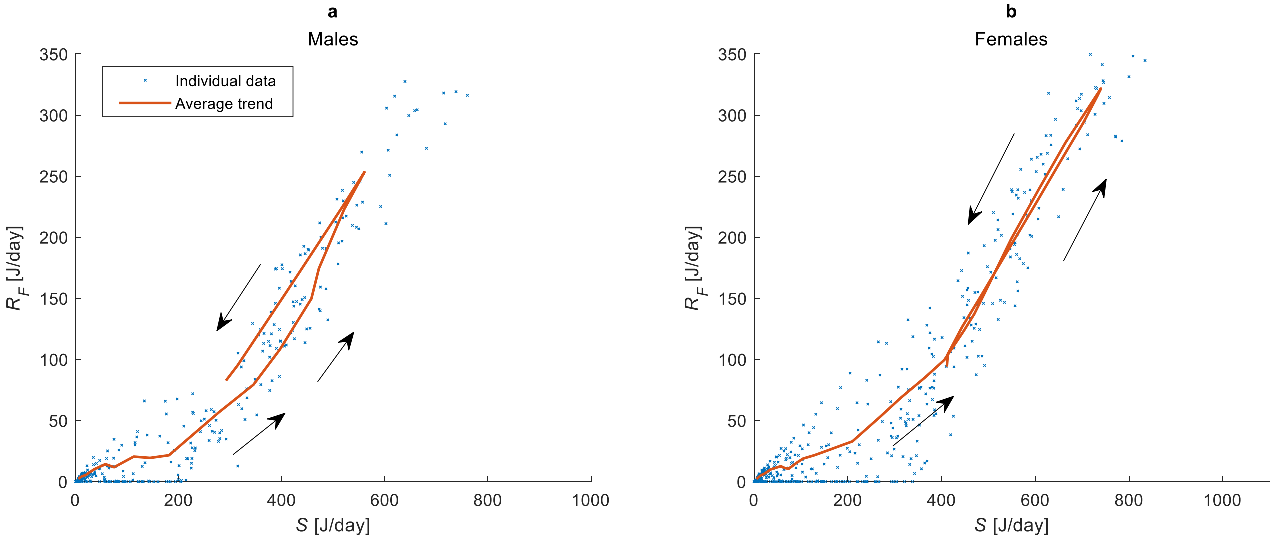

Fig SI10. Average trends of feeding costs R_F versus ingestion rate S for (**a**) male and (**b**) female house crickets reared under ad libitum conditions at 28.6 °C. Average trends (solid curves) are age-wise average values of individual empirical data. Arrows indicate the direction of time. Initially, S and R_F increase with age (and body size), but eventually they peak and start to decrease with age (and body size).

The graphs in Fig SI10 indicate that $R_F(S)$ may be approximated by a piece-wise linear function (where $R_F(S)$ seems to continue 'backwards' after the ingestion rate S has peaked according to the same linear relation as before the peak, particularly for females). For both sexes, a piece-wise linear function, divided into two intervals, was applied to describe $R_F(S)$:

$$R_F(S) = \begin{cases} k_{F1} \cdot S & , \quad S \leq S_1 \\ k_{F1} \cdot S_1 + k_{F2} \cdot (S - S_1) & , \quad S \geq S_1 \end{cases} . \tag{SI36}$$

The break point S_1 was identified for each individual from empirical data (R_F versus S) by using the Matlab routine *ischange* with the option '*linear*', which identifies changes in slope and intercept for a linear regime. A mixed linear model was applied for the interval $S \geq S_1$, using $R_{F1} = k_{F1} \cdot S_1$ and the slope k_{F2} as free parameters with random effects only on R_{F1} (lowest AIC). The slope in the interval $S \leq S_1$ was calculated as $k_{F1} = R_{F1}/ S_1$, with estimations of R_{F1} and S_1 inserted. The resulting fixed effects fits, with the shape of 'hockey sticks', are shown together with individual data in Fig SI11. Relative random effects on R_{F1} are shown in Fig SI12. Fixed effects and SREs of obtained model parameters (S_1, k_{F1}, k_{F2}) are presented in Table SI9.

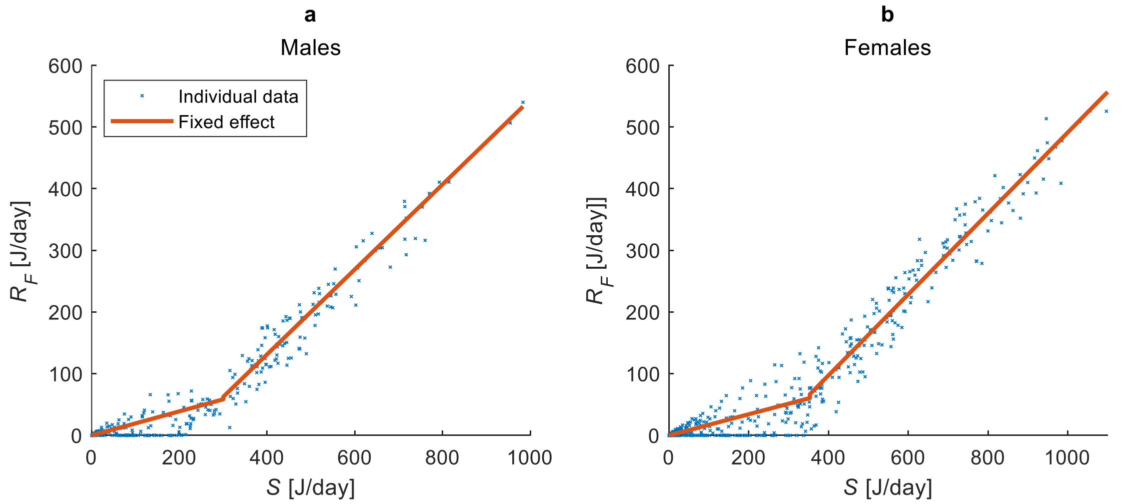

Fig SI11. Linear mixed effects model fit for feeding costs vs. ingestion rate for (**a**) male and (**b**) female house crickets reared under ad libitum conditions at 28.6 °C. Data points (x) represent individual empirical data of feeding costs R_F versus ingestion rate S. The fixed effects fit (solid lines) describes a piece-wise linear function with slopes k_{F1} and k_{F2} (Eq. (SI36)), estimated from a mixed linear model for interval $S \geq S_1$ with random effects on $R_{F1} = k_{F1} \cdot S_1$ (feeding costs at break point $S = S_1$). Males: R_{F1} = 63 ± 13 J/day (*SRE*: 10 %), S_1 = 300 ± 36 J/day, k_{F2} = 0.689 ± 0.023. Females: R_{F1} = 66 ± 13 J/day (*SRE*: 15 %), S_1 = 353 ± 36 J/day, k_{F2} = 0.656 ± 0.014.

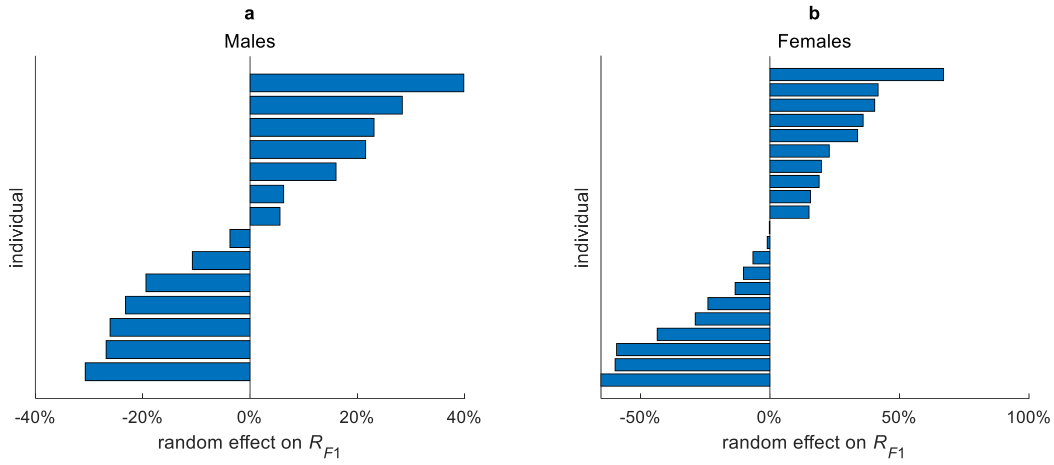

Fig SI12. Relative random effects of linear mixed effects model fit for feeding costs vs. ingestion rate for (**a**) male and (**b**) female house crickets reared under ad libitum conditions at 28.6 °C. Random effects on $R_{F1} = k_{F1} \cdot S_1$ (feeding costs at break point $S = S_1$) were obtained from a linear mixed effects model applied to data for feeding costs R_F versus ingestion rate S in the interval $S \geq S_1$. Plotted relative random effects are individual random effects normalised by division with corresponding fixed effect.

SI7.3. MODEL PARAMETERS OBTAINED FROM OPTIMIZATIONS

Results from model parameter estimations, using a non-linear optimization procedure to fit predicted and empirical growth curves, are presented in Table SI9 and Fig SI13. Corresponding fixed effects solutions are plotted with empirical growth data in Fig SI14. Individual solutions (accounting for random effects) are shown in Fig SI15. Notice that these growth trajectories (Figs SI14-15) were obtained by fitting Eq. (5) (an alternative formulation of MGM expressed in terms of resting metabolism and maintenance) to observed growth curves, while the results presented in Figs 3-4 were obtained by applying all MGM parameters in Table SI9 (estimated with various methods) to Eq. (4) (MGM as originally formulated in terms of ingestion, feeding costs and maintenance). As expected, model fits in Figs SI14-15 are better than in Figs 3-4.

By using both a_{NR} and a_{NS} as independent female parameters, abandoning the assumption of a single relative defence allocation normalisation constant for both somatic and reproductive tissue ($a_{NR} = a_{NS} = a_N$), it was not possible to obtain an accurate solution with *nlmefit* in Matlab. However, the 'inverse problem' methodology [21, 22] could be performed, resulting in fixed effects that were similar to the previously obtained value ($a_{NR} \approx a_{NS} \approx a_N$), supporting that $a_{NR} = a_{NS}$ is a reasonable assumption.

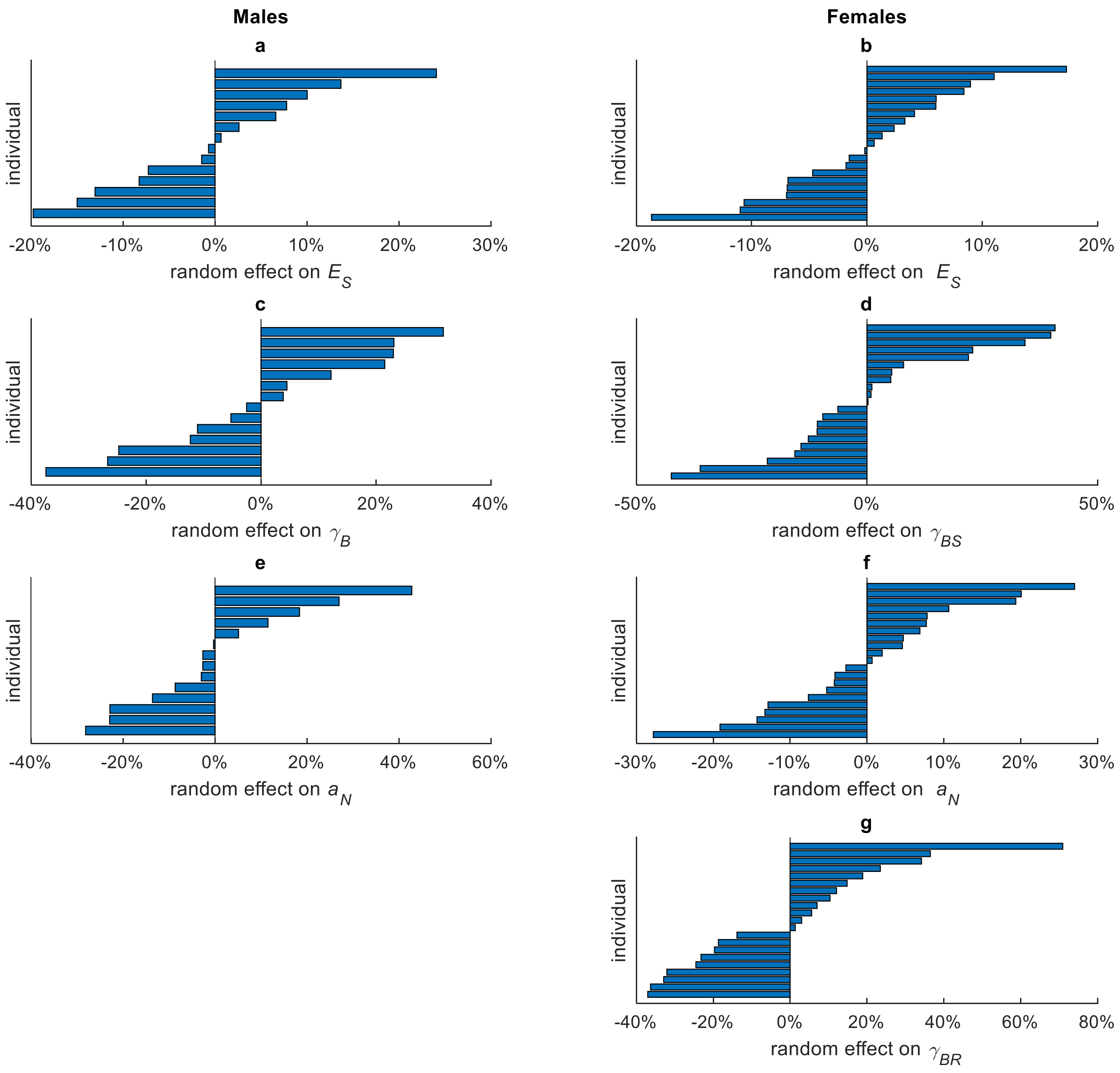

Fig SI13. Relative random effects for male (left column) and female (right column) model parameters, estimated by non-linear mixed effects model fits between predicted and empirical growth curves for house crickets reared under ad libitum conditions at 28.6 °C. Plotted relative random effects are individual random effects normalised by division with corresponding fixed effect. See Table SI9 for fixed effects and *SRE*s.

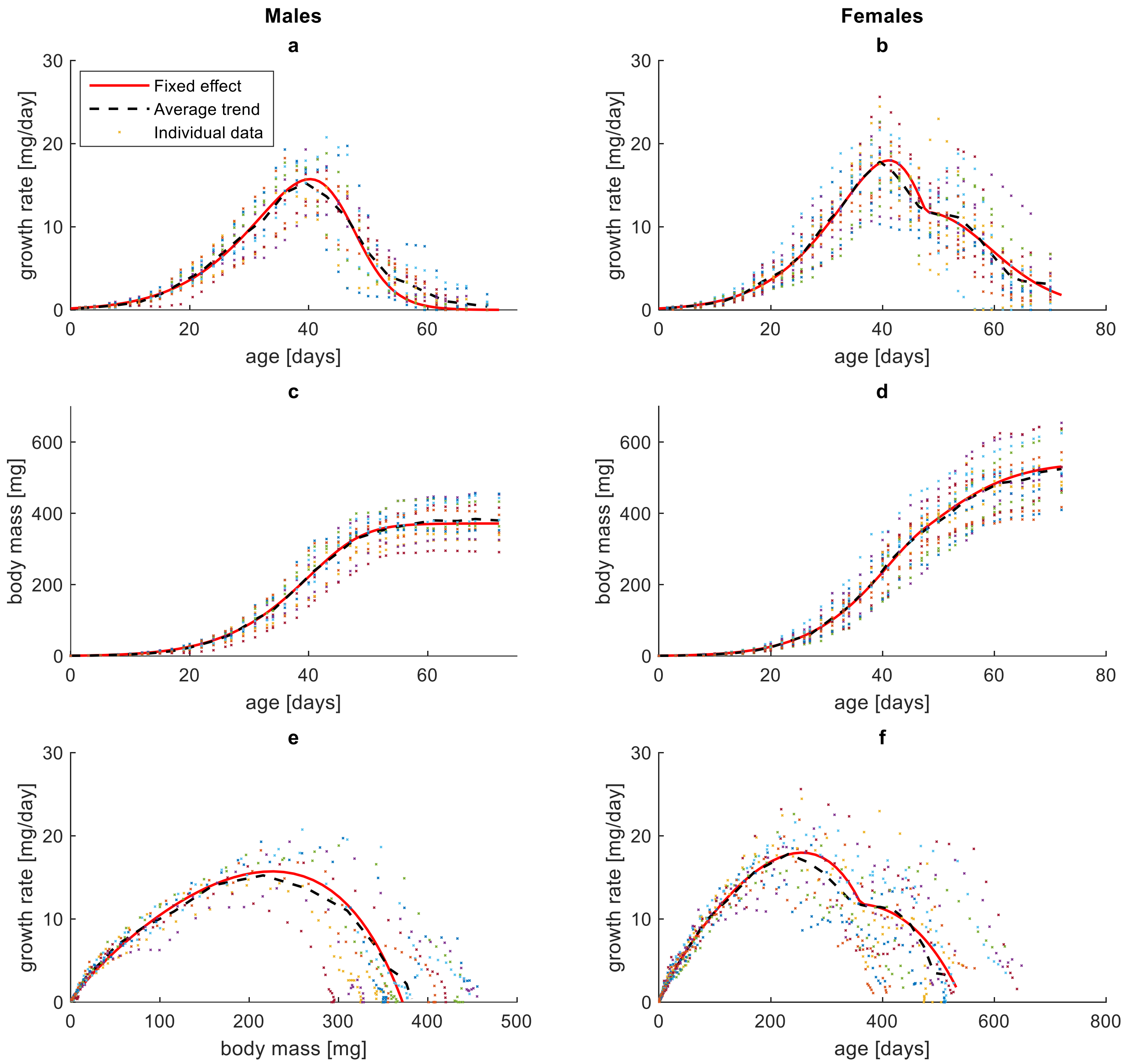

Fig SI14. Fixed effects solutions (solid curves) of growth rate, (dW/dt) and body mass (W), obtained from non-linear mixed effects model (applied to Eq. (5)), and empirical data for male (left column) and female (right column) house crickets reared under ad libitum conditions at 28.6 °C. Average trends (dashed curves) are age-wise average values of individual empirical data.

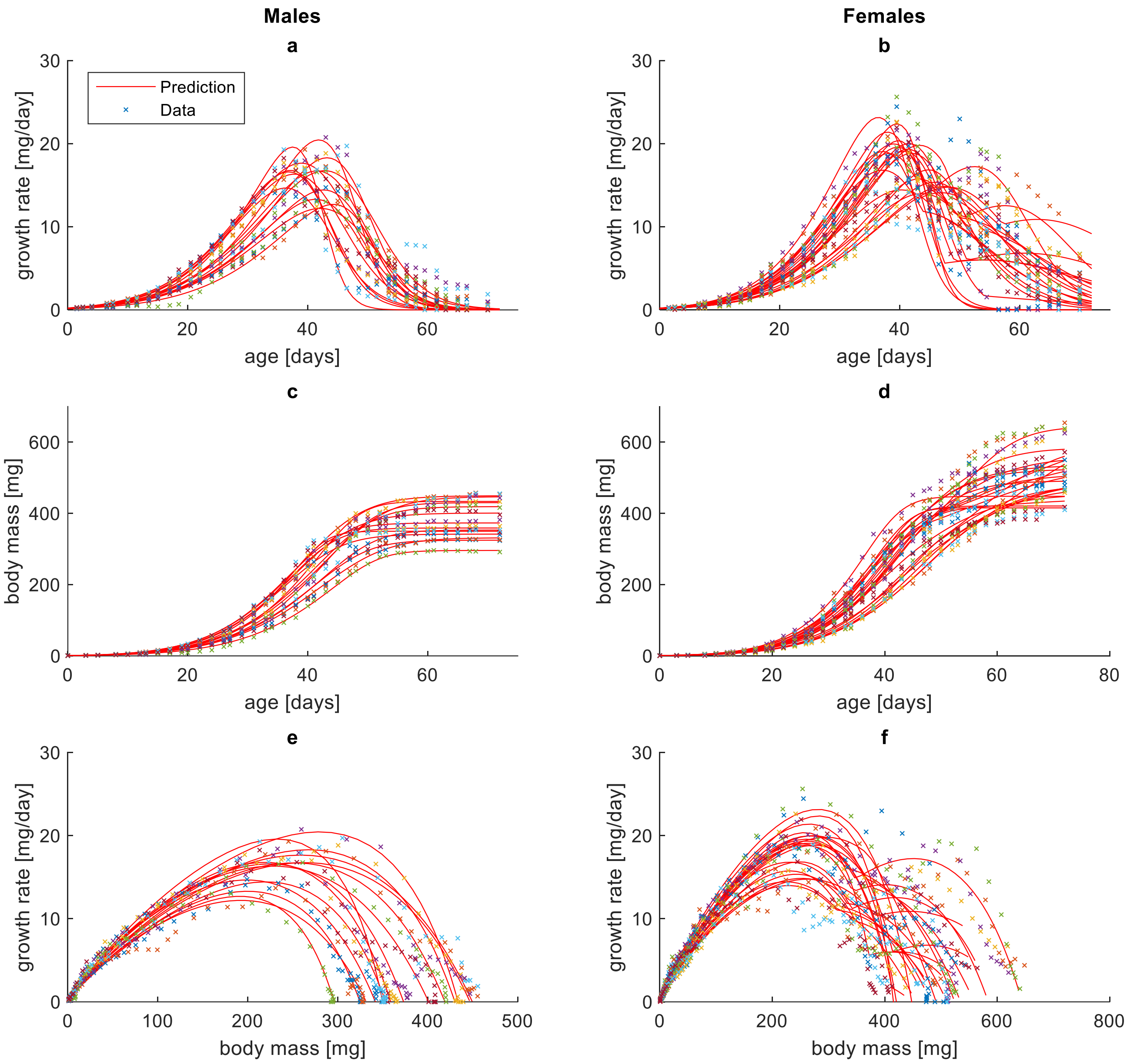

Fig SI15. Individual predictions (solid curves) for growth rate (dW/dt) and body mass (W), obtained from non-linear mixed effects model (applied to Eq. (5)), and individual empirical data for male (left column) and female (right column) house crickets reared under ad libitum conditions at 28.6 °C.

SI7.4. TOTAL METABOLIC RATE

Total metabolic rate R_{tot} versus body mass W was indirectly calculated from empirically collected data. The energy balance of a growing animal requires that assimilation rate ($A = eS$) equals the sum of total metabolic rate (R_{tot}) and the rate at which energy is bounded into synthesised biomass ($G = E_M \cdot dW/dt$). The total metabolic rate can thus be expressed in terms of the ingestion rate (S) and the growth rate (dW/dt), both measured in the experiment, using estimated values of assimilation efficiency (e) and biomass energy density (E_M):

$$R_{tot} = A - G = eS - E_M \frac{dW}{dt} . \quad \text{(SI37)}$$

An allometric relation was assumed for total metabolic rate ($R_{tot} = a_T \cdot W^{b_T}$) in the juvenile interval ($W \leq W_{mat}$) and a linear mixed effects model was applied to empirical data of $\log_{10}(R_{tot})$ versus $\log_{10}(W)$ for each sex, separately. A random effect (that varies by individual) was included for the intercept (not for the slope), thereby minimizing AIC. Since the exclusion of random effects for b_T explained data best, a small individual variation in the allometric exponent (b_T) was indicated. The results, including fixed and random effects of estimated parameters, are presented in Table SI8 and Fig SI16.

Table SI8. Fixed effects of allometric parameters for total metabolic rate (± 95 % CI) during ontogeny ($W < W_{mat}$) of house crickets reared under ad libitum conditions at 28.6 °C. Since confidence bounds are symmetric for $\log_{10}(a_T)$, they are asymmetric for the normalisation constant a_T. Standardized random effects (*SRE*) are included for $\log_{10}(a_T)$.

**Sex**	$\log_{10}(a_T)$	a_T [J/(day·mg^{b_T})]	b_T
Males	0.326 ± 0.082 (*SRE*: 28 %)	2.12 [+0.44/-0.36]	0.783 ± 0.036
Females	0.361 ± 0.084 (*SRE*: 38 %)	2.29 [+0.49/-0.40]	0.772 ± 0.032

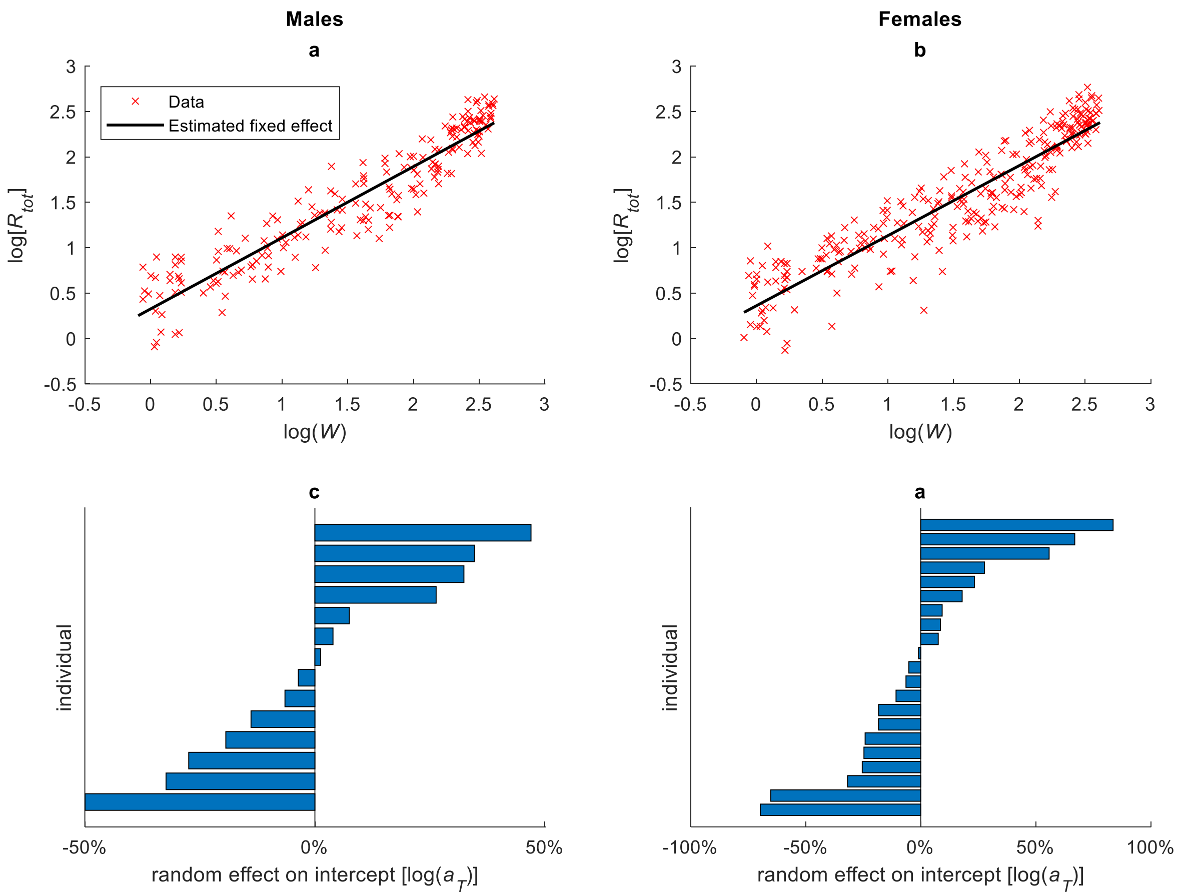

Fig SI16. Linear mixed effects model applied to empirical data for $\log_{10}(R_{tot})$ versus $\log_{10}(W)$ during ontogeny ($W \leq W_{mat}$), where R_{tot} is total metabolic rate (J/day) and W is body mass (mg) for male (left column) and female (right column) house crickets reared under ad libitum conditions at 28.6 °C. Random effects were included for the intercept. Residuals were checked to be rather evenly distributed. Plotted relative random effects are individual random effects normalised by division with corresponding fixed effect.

SI8. Results of model parameter estimations

Estimations of values of the parameters required to apply the version of MGM analysed here (Eq. (5)) to house crickets under ad libitum conditions was a four-step process where parameters were (*i*) obtained from literature (Note SI5), (*ii*) estimated directly from experimental data (Note SI7.1), (*iii*) estimated from experimental data with statistical methods (Note SI7.2), or (*iv*) estimated indirectly using ‘inverse model optimization’ (Note SI7.3). The results of all parameter estimations are summarized in Table SI9 (including fixed effects and standardized random effects (*SRE*s) where applicable).

Table SI9. Parameters for growth model of house crickets (*A. domesticus*) reared at 28.6 °C. Fixed effects (*FE*) with 95 % CI are presented for empirically estimated parameters. Standardized random effects (*SRE*) indicate individual variation in model parameters (Eq. (6)). The parameters α, k and S_1 are presented on a mass basis (as they were measured in the experiment). Before insertion into the growth model, these values must be converted to an energy basis by multiplication with the food energy density E_f = 13.4 J/mg. See Note SI5 for details on parameter estimations from literature and Note SI7 for details on parameter estimations from experimental data.

			Males		Females	
**Parameter**		**Unit**	*FE*	*SRE*	*FE*	*SRE*
Energy density of synthesised biomass [(1)]	E_M	J/mg	6.1	-	6.3	-
Assimilation efficiency [(1)]	e	-	0.75	-	0.75	-
Birth mass [(2)]	W_0	mg	0.8 ± 0.1	17 %	0.8 ± 0.1	17 %
Mass at maturity [(2)]	W_{mat}	mg	348 ± 24	12 %	368 ± 19	11 %
Ingestion rate normalisation constant [(3)]	α	$\frac{\text{mg}}{\text{mg}^{\beta}\cdot\text{day}}$	0.396 [+0.058/-0.050] [(5)]	19 % [(6)]	0.363 [+0.066/-0.056] [(5)]	34 % [(6)]
Ingestion rate allometric exponent [(3)]	β	-	0.757 ± 0.033	5 %	0.791 ± 0.035	8 %
Ingestion rate constant [(3)]	k	$\frac{\text{mg}}{\text{mg}^{1+\beta}\cdot\text{day}}$	0.039 ± 0.050	100 %	0.021 ± 0.005	11 %
Mass at maximal ingestion rate	W^*	mg	367 ± 25 [(2)]	20 %	428 ± 23 [(3)]	13 %
Ingestion rate at break point for feeding costs [(3)]	S_1	$\frac{\text{mg}}{\text{day}}$	22 ± 3	10 % [(7)]	26 ± 3	15 % [(7)]
Feeding costs slope (Interval 1) [(3)]	k_{F1}	-	0.195 ± 0.048		0.171 ± 0.047	
Feeding costs slope (Interval 2) [(3)]	k_{F2}	-	0.689 ± 0.023	-	0.656 ± 0.014	-
Specific growth overhead cost [(4)]	E_S	$\frac{\text{J}}{\text{mg}}$	2.79 ± 0.19	12 %	2.82 ± 0.13	9 %
Specific basal maintenance cost [(4)]	γ_B	$\frac{\text{J}}{\text{mg}\cdot\text{day}}$	0.136 ± 0.017	21 %	-	-
Specific somatic basal maintenance cost [(4)]	γ_{BS}	$\frac{\text{J}}{\text{mg}\cdot\text{day}}$	-	-	0.126 ± 0.015	23 %
Specific reproductive basal maintenance cost [(4)]	γ_{BR}	$\frac{\text{J}}{\text{mg}\cdot\text{day}}$	-	-	0.375 ± 0.055	28 %
Relative defence allocation normalisation constant [(4)]	a_N	mg^{-1}	0.0018 ± 0.0002	20 %	0.0016 ± 0.0001	14 %

Note: [(1):] Estimated from Woodring et al. [16] prior to experiments. [(2):] Calculated directly from experimental data. [(3):] Estimated from statistical model. [(4):] Estimated from 'inverse model optimization'. [(5):] Confidence bounds symmetric for $\log_{10}(\alpha)$ and hence asymmetric for α. [(6):] *SRE* on $\log_{10}(\alpha)$. [(7):] *SRE* on $R_{F1} = k_{F1}\cdot S_1$.

SI9. Supplementary discussion

SI9.1. GENERALITY OF MGM PARAMETERS

Individual variation in estimated model parameters of MGM (Table SI9) were measured by standardized random effects (*SRE*s, Eq. (6)). A small *SRE* is believed to indicate a robust species-specific parameter, represented by the fixed effect.

The observed average mass at maturity was somewhat larger for females (368 ± 19 mg) than for males (348 ± 24 mg), but the difference was not significant, and *SRE*s were intermediate (11-12 %). This is probably an important sex-specific life-history trait for the species and less individual variation makes sense.

The allometric exponent β for ingestion rate S during the juvenile phase ($S = \alpha W^{\beta}$) was the only MGM parameter with really small *SRE*s (5-8 %). Males (0.757 ± 0.033) had lower value than females (0.791 ± 0.035), but the difference was not significant. These exponents are somewhat (but not significantly) lower than $\beta = 0.820 \pm 0.031$, previously reported for ectothermic poikilotherms [15]. However, the values are higher than the allometric exponent for ad libitum ingestion rate of 2/3, applied by standard DEB models, which assumes that digestion and other food processing activities are limited by transports occurring through surfaces [23]. It has previously been suggested that $\beta = 2/3$ during the ontogeny of insects, whereas maximum ingestion rates scales to ultimate body mass with a 3/4 power exponent between insect species [24]. This is not supported by our data. The allometric exponent obtained here ($\beta \approx 0.8$) is probably a robust parameter for house crickets and it remains to be seen if it represents a more general value for other insects. The allometry may reflect a combination of size-dependent foraging behaviour and physical limitations of the gastrointestinal system. The latter may depend on fluxes through surfaces (indicating $\beta = 2/3$), but if the gastrointestinal system does not grow isometrically, as observed in some insects [25], this may cause the allometric exponent to be larger than 2/3 (as observed here).

Considerable individual differences in feeding behaviour are probably responsible for the large *SRE*s of the ingestion rate normalisation constant α for males (19 %) and females (34 %). Ingestion rate parameters in the post-mature interval ($dS/dW = k\ (W^{*}-W)/W^{1-\beta}$) had large *SRE*s for males (100 % for k, and 20 % for W^{*}), but only intermediate *SRE*s for females (11 % for k, and 13 % for W^{*}). Possibly,

feeding behaviour after maturity is more strictly regulated in females, who invest more in reproductive growth.

Parameters that relate feeding costs R_F to ingestion rate S, describing a piece-wise linear function with one breakpoint (S_1) and two slopes (k_{F1}, k_{F2}), had small to intermediate *SRE*s (10-15 % for $R_{F1} = k_{F1}S_1$ and no *SRE* for k_{F2}). Hence, increases in ingestion rate seem to affect feeding costs similarly across individuals.

The specific growth overhead cost E_S had fairly small *SRE*s (9-12 %) compared to other model parameters, except β. The fixed effect was similar for males and females, despite large differences in relative gonadal growth after maturity [16]. Thus, the estimated value of $E_S \approx 2.8$ J/mg may be a rather robust species-specific parameter.

Specific basal maintenance costs (γ_B, γ_{BS}, γ_{BR}), representing non-negotiable costs for maintenance per unit body mass, had fairly large *SRE*s (21-28 %), though large individual variation was not expected. Non-negotiable maintenance costs are processes that are necessary to keep the animal alive (such as maintaining ion potentials across cell membranes). These costs, viewed at the cellular level, are expected to be similar for similar-sized cells of similar type between individuals of the same species. However, if there are individual differences in body composition and growth strategy with respect to the relative contribution from cell enlargement and cell division, individual differences in specific basal maintenance costs are expected. Interestingly, parameter estimates of female specific basal maintenance costs were considerably larger for reproductive tissue than for somatic tissue ($\gamma_{BR} = 0.375 \pm 0.055$ J/(mg·day), $\gamma_{BS} = 0.126 \pm 0.015$ J/(mg·day)). Costlier maintenance of reproductive tissue may be a consequence of higher requirements due to larger complexity, higher priority due to larger effects on fitness or a combination of both.

The relative defence allocation normalisation constant a_N had *SRE*s that were large for males (20 %) and intermediate for females (14 %), but had no significant sex difference in fixed effects. For this parameter, some individual variation is expected, since it reflects a life-history trade-off between energy spent on growth and energy used for maintaining tissues in good shape. Different strategies may reflect individual genetical differences.

Although several parameters had large *SRE*s, all were dominated by fixed effects (*SRE*s well below 100 %), except for the male adult ingestion rate constant k (with $SRE \approx 100$ %). It thus seems reasonable to apply the estimated fixed effects as approximate constants in a general growth model for the species.

In this study, the growth model was calibrated to a specified fixed temperature. However, metabolic rates and growth rates generally increase with temperature [26]. Model parameters that describe rates (α, k, S_1, γ_B, γ_{BS}, γ_{BR}) are thus expected to be temperature-dependent. A simple way of scaling these parameters from one temperature to another is by multiplication with the Boltzmann-Arrhenius factor (the temperature-dependent factor of Eq. (1)), possibly using different activation energies for ingestion (α, k, S_1) and maintenance (γ_B, γ_{BS}, γ_{BR}), since they are different physiological processes. Also, mass at maturity (W_{mat}) is expected to be temperature-dependent, since the transition from nymph to adult can occur earlier and at a smaller body mass when the growth rate is higher.

SI9.2. Applied model simplifications

To enable model parameter estimations from an optimization procedure on the limited data set of the current study, a number of model simplifications were necessary (in order to reduce the number of free parameters). More specifically, this was achieved by assuming constant biomass energy density E_M, constant specific growth overhead cost E_S and constant mass-specific basal maintenance costs γ_{BS} and γ_{BR} (for somatic and reproductive tissue, respectively). Approximately constant E_M in house crickets is supported by data from Woodring et al. [16], see Note SI5.2. With E_M constant, also E_S must be constant to obtain a continuous growth rate at maturity, as indicated by collected data (Fig SI4f, Fig SI5i). Approximately constant body compositions before and after imago emergence, combined with constant E_S suggest that differences due to cell growth and cell division are of minor importance for house crickets. The adopted assumption of constant mass-specific basal maintenance cost for each individual thus seems reasonable.

The version of MGM applied here made the simplifying assumption that somatic and reproductive growth in females are separated in time. This is supported by other studies on house crickets [16], showing that female growth before imago emergence is almost entirely somatic (Note SI5.2). During early ontogeny, growth consists of synthesis of a protein-rich residual body and during late ontogeny, it is equally divided between continued residual growth and growth of a lipid-rich fat body (Table SI2).

The initial phase of the female imago stage is dominated by gonadal growth and has no net growth of somatic tissue, but some residual growth is equated by consumed fat body (Table SI3). The conversion of fat body into somatic tissue is not explicitly considered here, but the associated costs may be included in MGM as increased somatic maintenance costs after maturity.

SI9.3. INCLUSION OF ADDITIONAL EFFECTS

To capture observed growth patterns in an insect, the growth model (Eq. (SI10)) assumed increasing relative defence allocation (increasing mass-specific negotiable maintenance costs) with body mass (Eq. (SI23)). It was also tried to apply constant defence allocation ($b_{NS} = b_{NR} = b_N = 0$) and compensate for the simplification by including other effects (considered in more detail in Mauritsson and Jonsson [14]). These were; 1) increasing somatic biomass energy density E_{MS} due to changing composition of somatic tissue; 2) varying specific somatic growth overhead cost E_{SS} due to increasing contribution from cell enlargement to somatic growth; and 3) decreasing specific somatic basal maintenance cost γ_{BS} due to decreasing mass-specific cost of cell-surface-dependent maintenance processes. Neither of these additional effects (in combination with constant defence allocation) could generate accurate fits with empirical growth curves. It was concluded that increasing relative allocation to negotiable maintenance costs is an assumption that is required for describing observed growth patterns with the present formulation of MGM.

Inspection of the empirical average trend for female growth rate versus body mass (Fig SI5i), vaguely suggests that the curve may flatten once again (at a body mass of about 500 mg). This study lack data for the subsequent trajectory and further studies are required to investigate if there is indeed an additional break point due to female energy reallocations before ultimate body size is reached.

SI9.4. COMPARISON WITH OTHER GROWTH MODELS

MGM will here be compared with three other growth models; the ontogenetic growth model (OGM), which is a special case of the Generalized Standard Growth Model (GSGM, Eq. (2) with $d \leq 1$) and two models that deviate from GSGM; a modification of the standard DEB model, developed specially for insects, and a model proposed by Makarieva et al. [27], that has previously been shown to predict observed growth in an insect better than GSGM [14].

SI9.4.1. The ontogenetic growth model

A version of OGM for ad libitum conditions [8] assumed that resting metabolic rate R_R (including maintenance costs and growth overhead costs) follows an allometric relation ($R_R = R_M + R_G = aW^b$), that the specific growth overhead cost E_S is constant and that maintenance costs are proportional to body mass ($R_M = \gamma W$). By adding these assumptions to the MGM description of growth overhead costs ($R_G = E_S \cdot dW/dt$), the OGM growth equation can be written as:

$$\frac{dW}{dt} = \frac{1}{E_S}\left[R_R - R_M\right] = \frac{1}{E_S}\left[aW^b - \gamma W\right]. \qquad \text{(SI38)}$$

Unlike MGM (Eq. (SI10)), resting metabolism (instead of assimilation) is in OGM considered as the contribution process, while maintenance (instead of total metabolism) is the cost. Furthermore, and importantly, MGM does not assume a linear relation between maintenance and body mass. As previously demonstrated, a growth equation of this shape (Eq. (SI38)) cannot capture observed growth patterns in some insects [14].

Hou et al. [7] extended OGM to food restricted growth, assuming that the total metabolic rate (resting metabolic rate plus costs for feeding and other activities) is proportional to the resting metabolic rate ($R_{tot} = fR_R$), yielding the OGM growth equation expressed in terms of assimilation (A) as:

$$\frac{dW}{dt} = \frac{1}{E_M + fE_S}\left[A - fR_M\right] = \frac{1}{E_M + fE_S}\left[A - f\gamma W\right]. \qquad \text{(SI39)}$$

This formulation of OGM has similarities with the general formulation of MGM (Eq. (SI10)), but do not explicitly consider feeding costs and imposes a relation between total and resting metabolic rate that lacks mechanistic foundation and is also incompatible with regulation of maintenance in response to food limitation (as suggested by MGM). Assimilation under food restriction (as a function of age) is in OGM described as a specified fraction of corresponding assimilation under ad libitum conditions (as a function of age), calculated from the energy balance ($A = R_{tot} + G$) with insertion of the ad libitum body mass solution, obtained from Eq. (SI38). This is a rather cumbersome way of describing the level of food availability.

SI9.4.2. The insect DEB growth model

To better predict insect growth, Maino and Kearney [28] modified the standard DEB growth model by assuming increasing (instead of constant) surface-specific assimilation rate with increasing structural volume. Contrary to the standard DEB model, where reserve and structure increase proportionally under ad libitum conditions, this 'insect DEB growth model' predicts an increasing proportion of reserve, which implies increasing biomass energy density (since reserve has larger amounts of energy rich substances) and decreasing mass-specific maintenance (since reserve has no maintenance costs). The latter is the opposite prediction to MGM, where increasing relative allocation to negotiable maintenance results in increasing mass-specific total maintenance costs during growth. The modified DEB growth model was able to capture the near-exponential growth observed in many insects during early ontogeny [28], but failed to predict growth at later stages and could not accurately predict an asymptote. As an explanation, the authors suggested that insect growth is terminated by some other mechanism than mismatched scaling between resource supply (assimilation) and costs (maintenance), such as a developmental cue. MGM, on the other hand, predicts an asymptote.

The insect DEB growth equation does not have the same mathematical structure as GSGM (Eq. (2) with $d \leq 1$), but can be written on this form approximately (with $d < 1$ due to decreasing proportion of structure that requires maintenance). By fitting the growth equation to growth data from the literature, Maino and Kearney [28] approximated the allometric exponent of the assimilation rate for Orthoptera to $b \approx 0.892$ (to be compared with the allometric exponent for ingestion rate $\beta \approx 0.8$, here measured in house crickets). As previously demonstrated [14], GSGM (where $b < d \leq 1$) is unable to capture observed growth patterns from birth to ultimate size in house crickets, in accordance with the view of Maino and Kearney [28] that the insect DEB growth model is only valid for early stages of ontogeny.

SI9.4.3. The Makarieva growth model

As shown in Mauritsson and Jonsson [14], GSGM is unable to capture the hump-shaped curve that characterizes observed growth rate vs. body mass in male house crickets under ad libitum conditions (Fig SI5i), but a model for food-restricted growth developed by Makarieva et al. [27] may do it quite well (using four free parameters). Their model describes energy assimilated from food as distributed between metabolism and growth, assuming that energy expenses allocated to growth is a specified fraction of the assimilation rate, called the *growth efficiency* (ε), a function of body mass that takes

different shapes depending on food conditions. The Makarieva model considers all growth expenses as energy bounded into synthesised biomass and equates metabolism with resting metabolism, described by a power allometry. Assimilation is adjusted to meet metabolic demands under ad libitum conditions and consequently increases with body mass as long as the growth rate increases and for some period beyond, but eventually decreases with further growth and finally balances resting metabolism. This makes sense in view of the observed ingestion rates for house crickets, which peak and then decrease (Fig SI4c, Fig SI5f), but observed ingestion rates peak much later than observed growth rates (Fig SI4f, Fig SI5i), which is not predicted by the Makarieva model. Another deficiency of the Makarieva model is the neglection of growth overhead costs and activity expenses. Furthermore, a linearly decreasing growth efficiency with body mass (as investigated in Mauritsson and Jonsson [14]) will be too simplistic to capture the more complex growth patterns observed here in female house crickets (Fig SI4f, Fig SI5i).

MGM captures observed growth patterns by assuming increased relative allocation to negotiable maintenance costs during growth. An indirect outcome of this is a decrease in the fraction of assimilated energy that is available for growth, which can be interpreted as decreased growth efficiency with increased body mass.

SI9.5. FUTURE STUDIES

The new growth model (MGM) was here applied to house crickets growing under ad libitum conditions, but growth experiments on food-limited house crickets (*A. domesticus*) have also been performed and the data have been used to calibrate and evaluate MGM also for food-restricted growth [17]. A possible application of MGM is modelling of reaction norms for age and size at maturity under different levels of food limitation [29-31]. The model may also be applied beyond individual growth. More specifically, asymmetric competition and self-thinning in food restricted cohorts of house crickets may be further explored, previously considered by Jonsson [32].

References

1. Pütter, A., *Studien über physiologische Ähnlichkeit. VI. Wachstumsähnlichkeiten.* Pflügers Archiv für die gesamte Physiologie des Menschen und der Tiere, 1920. **180**: p. 298–340.
2. von Bertalanffy, L., *Quantitative laws in metabolism and growth.* Q Rev Biol, 1957. **32**: p. 217–231.
3. Pauly, D., *Gill size and temperature as governing factors in fish growth: a generalization of von Bertalanffy's growth formula*. 1979, Institut für Meereskunde PY, Kiel.
4. Kooijman, S.A.L.M., *Population dynamics on basis of budgets. The Dynamics of Physiologically Structured Populations*, in *Lecture Notes in Biomathematics*, J. Metz and O. Diekmann, Editors. 1986, Springer-Verlag: Berlin. p. 266-297.
5. Kooijman, S.A.L.M., *Quantitative aspects of metabolic organization: a discussion of concepts.* Philosophical Transactions of the Royal Society of London Series B-Biological Sciences, 2001. **356**(1407): p. 331-349.
6. van der Meer, J., *An introduction to Dynamic Energy Budget (DEB) models with special emphasis on parameter estimation.* Journal of Sea Research, 2006. **56**(2): p. 85-102.
7. Hou, C., K.M. Bolt, and A. Bergman, *A general model for ontogenetic growth under food restriction.* Proceedings of the Royal Society B-Biological Sciences, 2011. **278**(1720): p. 2881-2890.
8. Hou, C., et al., *Energy Uptake and Allocation During Ontogeny.* Science, 2008. **322**(5902): p. 736-739.
9. West, G.B., J.H. Brown, and B.J. Enquist, *A general model for ontogenetic growth.* Nature, 2001. **413**(6856): p. 628-631.
10. West, G.B., J.H. Brown, and B.J. Enquist, *Growth models based on first principles or phenomenology?* Functional Ecology, 2004. **18**(2): p. 188-196.
11. Gillooly, J.F., et al., *Effects of size and temperature on developmental time.* Nature, 2002. **417**(6884): p. 70-73.
12. Sibly, R.M. and J.H. Brown, *Toward a physiological explanation of juvenile growth curves.* Journal of Zoology, 2020. **311**(4): p. 286-290.
13. Zuo, W.Y., et al., *A general model for effects of temperature on ectotherm ontogenetic growth and development.* Proceedings of the Royal Society B-Biological Sciences, 2012. **279**(1734): p. 1840-1846.
14. Mauritsson, K. and T. Jonsson, *A new flexible model for maintenance and feeding expenses that improves description of individual growth in insects.* Scientific Reports, 2023. **13**(1).
15. Peters, R.H., *The ecological implications of body size*. 1983: Cambridge University Press.
16. Woodring, J., C.W. Clifford, and B.R. Beckman, *Food utilization and metabolic efficiency in larval and adult house crickets.* Journal of Insect Physiology, 1979. **25**(12): p. 903-912.
17. Mauritsson, K. and T. Jonsson, *A new mechanistic model for individual growth suggests upregulated maintenance costs when food is scarce in an insect.* Ecological Modelling, 2024. **491**.
18. Krüger, F., *Größenabhängigkeit des Sauerstoffverbrauches einheimischer Grillen.* Biologisches Zentralblatt, 1958. **77**: p. 581–588.
19. Gillooly, J.F., et al., *Effects of size and temperature on metabolic rate.* Science, 2001. **293**(5538): p. 2248-2251.

20. Hack, M.A., *The effects of mass and age on standard metabolic rate in house crickets.* Physiological Entomology, 1997. **22**(4): p. 325-331.
21. Banks, H.T., S. Hu, and W.C. Thompson, *Modeling and Inverse Problems in the Presence of Uncertainty*. 2014, Boca Raton, US: CRC Press.
22. Laubmeier, A.N., et al., *From theory to experimental design-Quantifying a trait-based theory of predator-prey dynamics.* Plos One, 2018. **13**(4).
23. Sousa, T., et al., *Dynamic energy budget theory restores coherence in biology.* Philosophical Transactions of the Royal Society B-Biological Sciences, 2010. **365**(1557): p. 3413-3428.
24. Maino, J.L. and M.R. Kearney, *Ontogenetic and interspecific scaling of consumption in insects.* Oikos, 2015. **124**(12): p. 1564-1570.
25. Blossman-Myer, B.L. and W.W. Burggren, *Metabolic Allometry during Development and Metamorphosis of the Silkworm Bombyx mori: Analyses, Patterns, and Mechanisms.* Physiological and Biochemical Zoology, 2010. **83**(2): p. 215-231.
26. Brown, J.H., et al., *Toward a metabolic theory of ecology.* Ecology, 2004. **85**(7): p. 1771-1789.
27. Makarieva, A.M., V.G. Gorshkov, and B.-L. Li, *Ontogenetic growth: models and theory.* Ecological Modelling, 2004. **176**(1-2): p. 15-26.
28. Maino, J.L. and M.R. Kearney, *Testing mechanistic models of growth in insects.* Proceedings of the Royal Society B-Biological Sciences, 2015. **282**(1819).
29. Nilsson-Ortman, V. and L. Rowe, *The evolution of developmental thresholds and reaction norms for age and size at maturity.* Proceedings of the National Academy of Sciences of the United States of America, 2021. **118**(7).
30. Plaistow, S.J., et al., *Age and size at maturity: sex, environmental variability and developmental thresholds.* Proceedings of the Royal Society B-Biological Sciences, 2004. **271**(1542): p. 919-924.
31. Day, T. and L. Rowe, *Developmental thresholds and the evolution of reaction norms for age and size at life-history transitions.* American Naturalist, 2002. **159**(4): p. 338-350.
32. Jonsson, T., *Metabolic theory predicts animal self-thinning.* Journal of Animal Ecology, 2017. **86**(3): p. 645-653.
